# Coupled Geophysical and Technoeconomic Industrial Growth Constraints on Geological Carbon Storage Scale-Up: Application to UK Offshore Resources

**DOI:** 10.1021/acs.est.6c10305

**Published:** 2026-11-02

**Authors:** Iman R. Kivi, Xiaowei Gao, Samuel Krevor

**Affiliations:** † Global Change Research Group, Mediterranean Institute for Advanced Studies, 541804 University of the Balearic Islands, Esporles, Balearic Islands 07190, Spain; ‡ Department of Earth Science and Engineering, 4615 Imperial College London, London SW7 2AZ, U.K.

**Keywords:** Climate change mitigation, Growth modeling, Geophysical constraints, Simplified-physics modeling, United Kingdom decarbonization roadmap, Storage resource

## Abstract

Geological carbon dioxide storage is central to meeting many national climate change commitments, but large-scale deployment may be limited by both techno-economic rates of growth and physics-based processes imposed by subsurface geology. We develop a modeling framework for the assessment of feasible CO_2_ storage scale-up trajectories that unifies constraints arising from industrial rates of growth, dynamic injection rate limits from reservoir pressurization, and static maximum cumulative storage resource. We employ logistic and Gompertz models to represent policy-compliant deployment trajectories, parametrized by the total cumulative storage resource. We then use a simplified physics model to estimate the resulting regional subsurface pressurization along these trajectories. Growth curves where reservoir pressurization exceeds safe limits are updated by reducing the static storage resource until residing in the safe deployment range. The result is both a growth trajectory and static storage resource achievable subject to injectivity limits. Applying the framework to the United Kingdom (UK), we find that offshore storage resources are sufficient to accommodate all government midcentury deployment rate scenarios up to 175 Mt yr^–1^. Cumulative storage of up to 78 Gt, representing a reference estimate of the UK storage resource base used in national planning, can be achieved under Gompertz growth trajectories. The more aggressive logistic trajectories are constrained by reservoir pressure, limiting the achievable resource base to 69 Gt. The framework is transferable to other regions to assess the feasibility of policy-driven CO_2_ storage pathways under coupled industrial growth and geophysical constraints, informing national decarbonization planning and global integrated assessment models.

## Graphical abstract

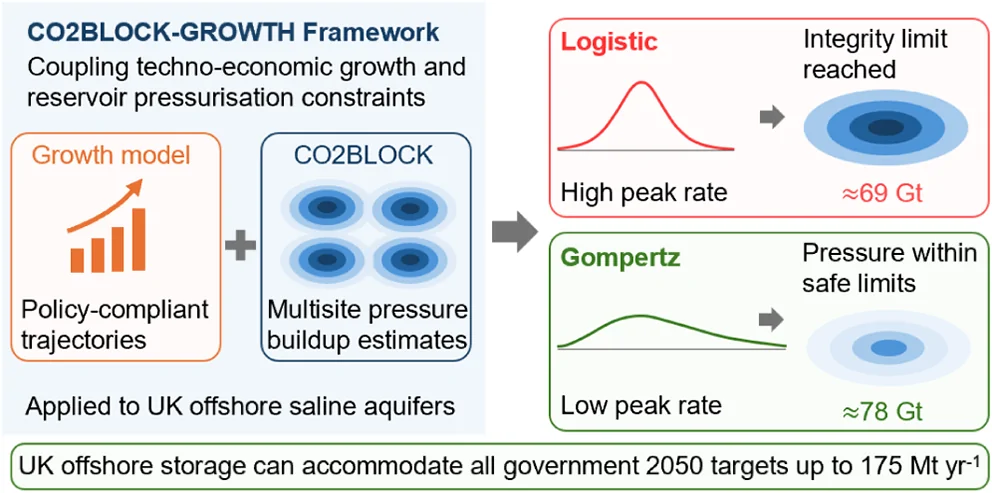

## 1 Introduction

The majority of climate change mitigation pathways require large-scale deployment of carbon dioxide storage in deep geological formations.^1–5^ Scenarios compiled by the Intergovernmental Panel on Climate Change (IPCC) that limit global warming to below 2 °C project annual subsurface CO_2_ injection rates of several billion tones (gigatonnes, Gt) by midcentury.^2^ The United Kingdom (UK) similarly incorporates geological CO_2_ storage as a central component of its decarbonization strategy, targeting storage rates of 20–30 Megatonnes (Mt) CO_2_ yr^–1^ by 2030 and 75–175 Mt CO_2_ yr^–1^ by 2050.^6^ The UK is uniquely positioned to plan such large-scale storage given its abundant offshore resources in the North Sea, which offer the potential to serve as a regional CO_2_ storage hub for Europe.^7^ The envisioned storage rates are comparable to the mass throughput associated with large-scale subsurface fluid handling in the UK hydrocarbon industry – nearly 57 Mt of oil equivalent were produced from the UK sector of the North Sea in 2023 ^8^ – underscoring the scale of industrial development required for CO_2_ storage to meet UK national climate targets.

Integrated assessment models are the primary tools used to project CO_2_ storage deployment within climate mitigation pathways.^2^ These models combine knowledge from different domains to to explore interactions between climate, economy, energy systems, and mitigation options.^9,10^ In these modeling frameworks, the CO_2_ storage contribution to emission abatement is typically determined through technoeconomic optimization of energy systems, with relative technology costs primarily influencing the deployment of competing mitigation options.^11^ Some modeling frameworks may impose global upper bounds on available storage capacity or annual injection rates, thereby incorporating a simplified representation of geophysical limits.^12–15^ Storage resource estimates used in integrated assessment models are commonly based on volumetric evaluations of rock pore space, combined with a storage efficiency factor to approximate the effects of CO_2_ flow and trapping dynamics underground.^16^ Such approaches sometimes fail to accurately capture these dynamics alongside reservoir pressurization and the geomechanical response of the storage complex, which collectively limit storage resource use.^17^ Accounting for these factors may substantially impact estimates of both ultimate storage capacities and sustainable injection rates, since excess reservoir pressure buildup may threaten storage complex integrity or induce earthquakes,^18,19^ and migrating CO_2_ plumes may elevate leakage risk if they intersect legacy wells and permeable faults.^20,21^

Beyond geophysical uncertainties in storage resource use, another critical factor that has remained underrepresented in integrated assessment models is a realistic representation of the sustained development rates at which CO_2_ storage deployment can occur.^15,22–26^ Globally, CO_2_ storage deployment is experiencing rapid growth, resembling the early stage expansion of large-scale energy infrastructure such as the petroleum industry in the early- to mid-20th century.^27–29^ Yet, historical industrial analogues show that such rapid growth is rarely sustained over the decades in the way projected by some integrated assessment models.^30,31^ Indeed, integrated assessment models have traditionally underrepresented key techno-socio-economic constraints on CO_2_ storage scaleup, including project development timelines, siting, property rights, regulatory approvals, financing and public acceptance.^32,33^ Thus, the feasibility of delivering geological CO_2_ storage at scales implied by climate targets remains highly uncertain, hinging on the interplay between achievable growth rates and the physical limits to safe injection.

To study CO_2_ storage scaleup challenges a number of scalable modeling frameworks have been developed. For the representation of techno-economic limitations, logistic growth models, originally developed to describe the expansion of extractive resource technologies,^34,35^ provide a useful means to capture the dynamics of CO_2_ storage.^15,22,23,26^ For the representation of geophysical processes, simplified-physics models have been developed to capture reservoir pressurization and CO_2_ plume migration underground over regional scales.^17,18,36,37^ Combining these two modeling frameworks enables an analysis of the temporal limits on the scaleup of geological CO_2_ storage. Szulczewski et al. (2012)^17^ develop a physics-based storage supply curve for the United States, accounting for constraints imposed by CO_2_ trapping and reservoir pressurization, and compared it with storage demand trajectories based on prescribed linear growth and decline in CO_2_ production. Their approach demonstrates how geophysical constraints influence the duration over which storage demand can be met, although it does not explicitly consider constraints on the growth of CO_2_ storage infrastructure. Fuhrman et al. (2025)^22^ show that when implementing combined growth and maximum total injectivity constraints into an integrated assessment modeling framework, the CO_2_ storage deployment is globally reduced. However, their regional injection-rate constraints are derived from storage supply curves and historical oil and gas production analogues, rather than explicitly modeling the evolution of reservoir pressurization under deployment trajectories. Combining technological growth constraints from the rate of injection well construction with pressure constraints on geological CO_2_ storage scaleup for the UK, de Jonge-Anderson et al. (2026)^38^ find that the rate of well construction is the limiting constraint on scaleup relative to projected mitigation pathways. However, their approach represents deployment through logistic growth in the number of wells, assuming fixed injection rates and operational lifetimes for individual wells. These studies demonstrate the importance of accounting for both deployment and geophysical constraints on scaleup trajectories and the cumulative storage resources that can ultimately be accessed. However, further investigation is needed to improve the representation of these constraints and their integration.

In this paper, we advance the identification of feasible scaleup trajectories for CO_2_ storage by unifying models of technological and both static and dynamic geophysical constraints into a single framework applicable anywhere around the world where sufficient data is available. In our framework, growth models (symmetric and asymmetric logistic curves) represent the techno-economic limits to CO_2_ storage deployment subject to static geological boundaries of the maximum total storage resource. Simplified physics models of reservoir pressurization are used to identify those growth trajectories that can be achieved without unsafe pressurization of the storage system. Our framework is an advance over existing approaches^17,22,38^ in its unification of static and dynamic geophysical constraints with techno-economic growth models. The framework also resolves a number of issues arising from the representation of techno-economic growth patterns in CO_2_ storage; the use of the static storage resource bases eliminates the arbitrary selection of saturation points in growth curves; The use of logistic growth curves are more flexible and broadly applicable than the use of a single empirical data set, e.g., of well construction or historical oil and gas production. We show how a single storage resource estimate arises naturally from the framework, identifying the resource base that can realistically be achieved under plausible growth trajectories, over time scales of relevance to climate change mitigation roadmaps. Applying this framework to the United Kingdom, we find that scaleup is limited not by the size of the storage resource, but in achieving the rates of industrial expansion required to deliver these deployment pathways.

## 2 Methodology

### 2.1 Growth Modeling Framework

To bridge the gap between near-term growth rates of geological carbon storage and long-term geophysical limits given finite subsurface storage resources, we employ phenomenological S-curve growth models to construct a feasibility envelope for CO_2_ storage deployment in the UK. This approach is grounded in the historical evidence of analogous extractive and energy industries, where sigmoidal growth functions have proven robust in characterizing production lifecycles constrained by finite resources.^27,34,39^

Adapting this framework to geological carbon storage, we build upon the methodology established by Zahasky and Krevor (2020)^26^ and its application for the UK and global scaling studies.^7,15^ Those studies relied on the symmetric logistic model, in which the instantaneous growth rate is proportional to both the cumulative quantity already developed and the fraction of the finite resource remaining. Growth is approximately exponential during the initial stage, when resource limitation is negligible, but progressively slows as the cumulative quantity approaches its asymptotic limit.^27,39^ In this model, the constraining effect of finite resource availability increases linearly with utiliation. As a result, the maximum growth rate occurs at exactly half of the asymptotic limit, yielding the characteristic symmetry of the logistic trajectory, which has a rate of decline that mirrors the rate of growth.

To capture the potential asymmetry in industrial scaleup, where the pace of infrastructure decommissioning or stabilization may differ from that of initial expansion, we extend this framework by also using the Gompertz growth model, in which resource limitation evolves nonlinearly as development progresses.^40^ Consequently, the maximum growth rate occurs at an earlier fraction of total resource utilization, leaving a larger fraction to be utilized more gradually after the peak. This feature generalizes the logistic model to nonsymmetric deployment trajectories. Drawing on analogue analyses of wind power and other energy technologies,^41,42^ this dual-model approach enables the construction of a feasibility envelope that encompasses growth trajectories with historical precedent.

In the logistic formulation, the cumulative storage trajectory *P*(*t*) and its corresponding injection rate *Q*(*t*) are defined as

1 $$P\left(t\right)=\frac{C}{1+\exp\left\{r\left(t_{p}-t\right)\right\}}$$

2 $$Q\left(t\right)=\frac{C\cdot r\cdot \exp\left(r\left(t_{p}-t\right)\right)}{(1+\exp{\left(r\left(t_{p}-t\right)\right)}^{2}}$$

where *C* (Gt) is an asymptotic cumulative storage bound, *r* (yr^–1^) is intrinsic growth constant that controls the curvature, i.e., the steepness, of the scaleup trajectory, *t*_*p*_ (yr) is the year at which *Q*(*t*) reaches its maximum peak rate. The inflection point of the rate curve on the growth limb occurs at (Figure 1)­

3 $$t_{n}=t_{p}-\frac{\ln\left(2+\surd 3\right)}{r}$$

**Figure 1:**
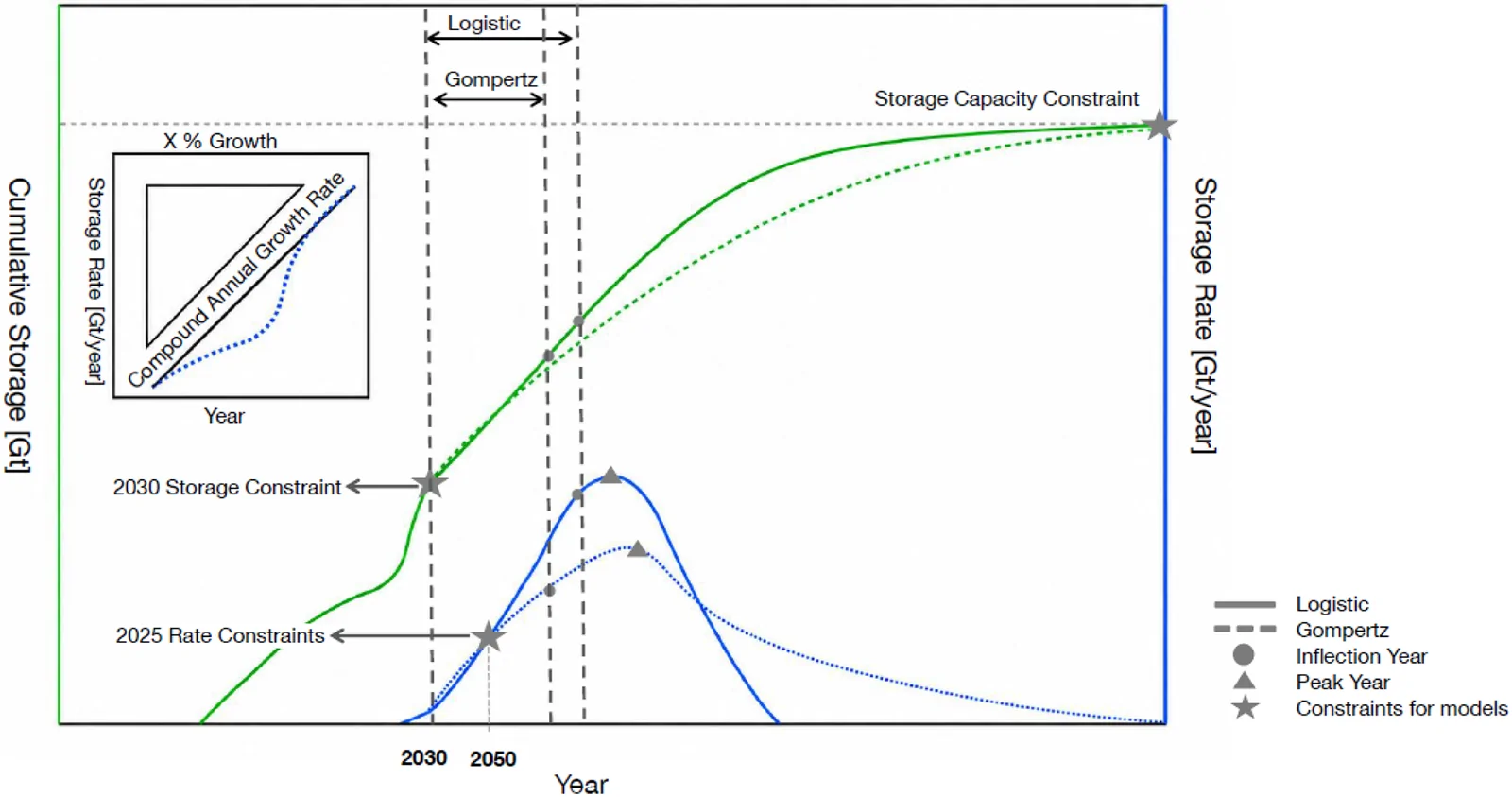
Schematic illustration of the logistic and Gompertz growth models for geologic carbon storage. Green curves represent cumulative CO_2_ storage, P­(t), referenced to the left-hand *y*-axis; blue curves represent annual CO_2_ injection rate, Q­(t), referenced to the right-hand *y*-axis. Solid and broken lines denote the logistic and Gompertz models, respectively. The two marked years by stars identify the prescribed model constraints, i.e., cumulative storage by 2030 and annual injection rate in 2050. The horizontal dotted line indicates the asymptotic storage resource base, C. Circles identify the inflection year, t_n_, of the injection-rate curves and the corresponding positions on the cumulative-storage curves; triangles identify the peak injection year, $t_{p}$ . Horizontal double-headed arrows indicate the intervals from 2030 to each model’s t_n_. The upper-left inset schematically illustrates growth in annual injection rate over time. The whole diagram is conceptual and is not drawn to scale.

This parameter provides model-internal marker for the end of the strongly accelerating, near-exponential build-out phase.

To represent bounded but nonsymmetric scaleup shapes, a Gompertz growth trajectory is formulated using its characteristic double-exponential form as

4 $$P\left(t\right)=C\exp\left[-\exp\left\{r\left(t_{p}-t\right)\right\}\right]$$

5 $$Q\left(t\right)=C\cdot r\cdot \exp\left(r\left(t_{p}-t\right)\right)\cdot \exp\left[-\exp\left\{r\left(t_{p}-t\right)\right\}\right]$$

6 $$t_{n}=t_{p}-\frac{\ln\left[\left(3+\sqrt{5}\right)/2\right]}{r}$$

Both of these models are constrained by the same three parameters (C, r , and *t*_*p*_ in eqs 1 and 2). The key difference is that the symmetric curves have a higher peak in the storage rate at an earlier time (Figure 1). For an equal storage resource base, the symmetric logistic curve will have trajectories that reach higher peak injection rates that are more likely to fall afoul of geophysical limits as compared with the asymmetric Gompertz curves. From that perspective, the use of symmetric logistic curves results in conservative outcomes for the assessment of the feasibility of storage rate targets. In other words, if a storage rate target is identified as feasible along a symmetric logistic trajectory, it will also be feasible along the Gompertz trajectory with the same parametrization – growth rate, 2050 target, and total resource base.

With both of the growth models, while r is the intrinsic shape parameter used to compute *t*_*n*_ and *t*_*p*_, it is not necessarily equal to an empirically interpretable percentage growth per year for deployment. To describe the effective early phase scaleup rate in a way that is comparable across models, we therefore define a compound annual growth rate, g, for cumulative storage over an explicitly defined early interval [t_0_, t_n_] as

7 $$g_{P\left[t_{0},t_{n}\right]}={\left(\frac{P_{\left(t_{n}\right)}}{P_{\left(t_{0}\right)}}\right)}^{1/\left(t_{n}-t_{0}\right)}-1$$

As such, g is used as a descriptive metric of pre-t_n_ build-out intensity, whereas r remains the model parameter that governs curvature and enables consistent calculation of timing metrics (including t_n_).

These *S*-curve models represent growth resulting from the aggregate affect of specific policy instruments, market incentives, financing conditions, engineering activities, and regulatory processes, but in this work we do not identify these specific contributions. Instead, the logistic models provide simplified and smoothed (averaged) scaleup trajectories, with empirical basis in past industrial developments, and representative of the aggregate impact of these processes on the deployment.^7^ Therefore, the growth trajectories should be interpreted as plausible deployment scenarios rather than predictions derived from explicitly modeling the underlying techno-economic mechanisms.

### 2.2 Parameters for the Growth Models

The models in eqs 1 and 2 are controlled by the parameters C, r, and t_p_. We parametrize C directly as described below, but parametrize the models with two other constraints that are of practical interest, and then solve for r and t_p_. First, we treat cumulative storage by 2030 as the takeoff point for the UK scaleup pathways. Accordingly, we set P(2030) equal to 0.3085 Gt CO_2_, derived from the UK project pipeline capacity.^43^ To bound cumulative storage, we set the geological capacity to C = 78 Gt CO_2_. This value is consistent with UK-wide storage assessments and is also used as a headline estimate in UK Government communications on offshore CO_2_ storage potential.^44^ Note that the parameter C represents a static geophysical constraint and is the maximum cumulative storage that can occur. However, the dynamic geophysical constraints arise from the distinct physics model, described in detail in Section 2.3. Thus, the parameter C defines the reference scaleup trajectories based on independent resource assessments. Where dynamic geophysical constraints prevent the prescribed resource base from being achieved, alternative trajectories can be generated in which C is no longer imposed. Instead, the achievable peak injection rate is constrained to allow the corresponding cumulative storage resource to emerge as a consequence of the growth model. As a result, the ultimate attainable CO_2_ storage scale identified by the coupled geophysical and growth constraints is independent of the initial resource-base estimate. Finally, a policy-derived constraint is imposed on the storage rate in 2050. We consider three alternative midcentury targets Q_2050_ equal to 75, 130, and 175 Mt CO_2_ yr^–1^, consistent with the range reported in the UK Climate Change Committee’s Net Zero advice.^45^ Each value is treated as a separate scenario, allowing the implied scaleup requirements to be compared under the same capacity ceiling and near-term takeoff point.

For each Q_2050_ scenario, we numerically solve for r and t_p_ by requiring the model to satisfy both anchors simultaneously. We then impose a timing constraint used in recent feasibility-envelope studies by requiring the peak year to occur no earlier than midcentury, i.e, t_p_ ≥ 2050.^7^ This condition keeps the 2050 rate targets on the rising limb of the trajectory and avoids calibrations that would imply an early onset of decline within the period where the analysis is intended to represent sustained scaleup. Allowing the peak to occur before 2050 would represent a fundamentally different deployment pathway in which storage has already entered decline by midcentury and, particularly for the symmetric logistic model, may demand unrealistically large peak injection rates to satisfy the prescribed resource base. The constraint also reflects the practical difficulty in the creation of a business environment that would enable the exploition of a natural resource close to the extent of the resource base. The resulting trajectories should thus be interpreted as accurate representations of multidecadal averaged pathways, but do not reflect the short-term (1–5 year) fluctuations that occur within any industry.

### 2.3 Dynamic Geophysical Constraints

Subsurface pressure buildup represents the main dynamic geophysical constraint on regional CO_2_ storage implementation and is thus the main focus of this modeling framework. Thus, in the second stage of our modeling framework we assess whether the projected CO_2_ storage scaleup trajectories in the UK derived from the logistic and Gompertz growth models (Section 2.2) are limited by basin-wide pressurization. To carry out this analysis we have made developments to the analytical tool CO2BLOCK^18,46^ (see Section S1 in Supporting Information for detailed explanation). We combine CO2BLOCK with the growth models to allocate the estimated storage resources under geophysical constraints to the projected CO_2_ storage scaleup trajectories in the UK. This modeling framework is named CO2BLOCK-GROWTH and is publicly available as open source software with the link provided under the data availability statement.

This dynamic resource allocation problem is governed by (1) the characteristic shape of the symmetric and asymmetric growth trajectories, featuring an increase over time, reaching a peak, and subsequently declining, and (2) the effective storage capacity of each geological unit, which depends on the injection duration and the associated subsurface pressure dynamics. To comply with these constraints, we implement a sequential allocation procedure and obtain end member results by evaluating the use of storage units in either descending or ascending order of capacity, discussed in detail below. For example, in the descending approach, the geological unit with the largest estimated storage resource is assigned first and remains active throughout the entire modeled period.

The allocation logic involves finding in consecutive steps a constant injection rate such that the injected CO_2_ mass matches that targeted by the growth trajectory (Figure 2). This approach relies on the principle that if the required volume can be injected at a constant rate, it can also be addressed under variable but lower rates of scaleup trajectories over longer durations, as the maximum pressure buildup attenuates with time. The remaining part of the injection trajectory is then matched by successively assigning the next units until fully covering the area beneath the injection rate curve, representing the total projected CO_2_ mass.

**Figure 2:**
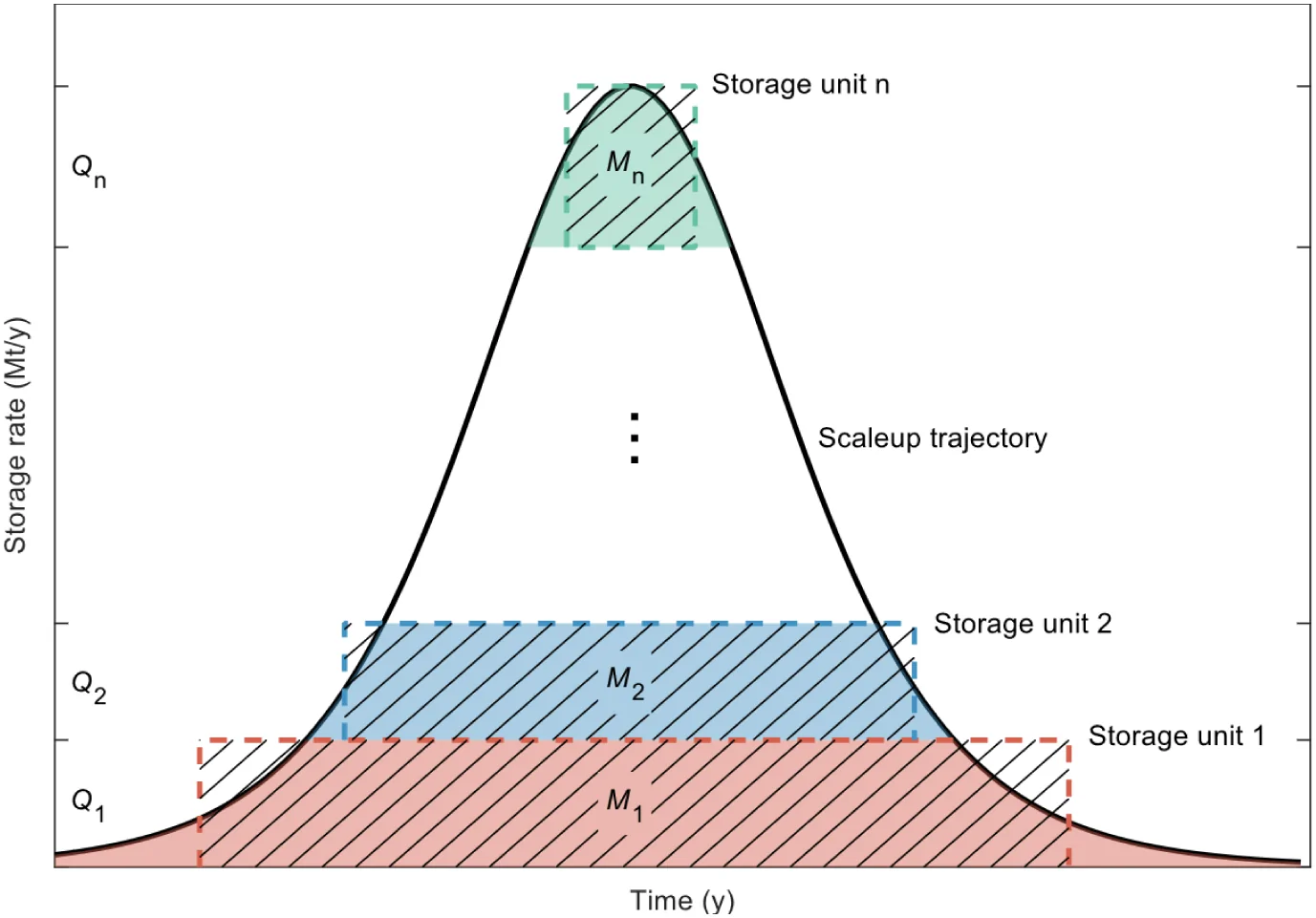
Schematic illustration of the workflow for simulating the use of CO_2_ storage resources along scaleup trajectories. The growth curve (in this case a symmetric logistic curve) serves as an envelope trajectory. Injection into individual geological units are then simulated at a constant maximum, across an optimum site number and spacing, where reservoir pressure does not exceed safe limits for rock failure and seismicity over the time period of consideration. Storage units are added such that the sum total CO_2_ mass *M* (sum of the hatched surfaces) equals the value prescribed by the scaleup trajectory.

Real world development scenarios are unknown and will exhibit some convolution of spatial organization, e.g., due to locations of CO_2_ transportation infrastructure and high quality storage resources, and random distribution reflecting the outcomes of market and policy driven processes like contract and license awards. To avoid arbitrary decision making in our modeling as mentioned earlier we apply two allocation strategies that are unrealistic in their spatial development of storage resources, but that represent end-member scenarios in terms of the geophysical limitations. Thus, these scenarios will encompass the range of plausible real world development scenarios insofar as injectivity limitations are concerned.

The first adopted resource allocation strategy represents a geophysically favorable configuration, where locations are developed in order of decreasing capacity. The first storage units have the largest permeability, areal extent and thickness, minimizing rates of pressure buildup. We also refer to this as the reference allocation strategy. The second end member scenario is one in which the resources are allocated in order of ascending capacity, i.e., the least favorable locations are developed first. We demonstrate that these are indeed end-member scenarios by also simulating random allocations of the storage resources. For this analysis, we generate 100 random sequence of the 27 storage units and independently apply the resource-allocation algorithm to each sequence.

Uncertainties also arise from the feasibility of developing a sufficient number of injection sites to fully utilize storage resources. It should be noted that each site may, in practice, comprise multiple injection wells located within a few kilometers from each other, but their pressure effects can reasonably be aggregated into a representative injection point in our modeling framework.^18,36^ The number of injection sites are primarily governed by the flow properties of the storage aquifers, which modulate pressure changes and may vary from one region to another. We also apply a minimum per site injection rate of 1 Mt yr^–1^ for economic viability of site development and a maximum of 20 Mt yr^–1^ acknowledging potential technological limitations.

To address site construction constraints, we draw an analogy with historical wellbore drilling for hydrocarbon production in the studied regions, providing a realistic benchmark for the number of wells that can be drilled within projected injection time frames. We spatially correlate more than 13,000 hydrocarbon wellbores in the UK Continental Shelf^47^ with candidate CO_2_ storage units, considering both their geographic distribution and target geological formations. This correlation provides an estimate of the number of wellbores uniquely associated with each storage unit (see the distribution in Figure S3). We translate this into a corresponding number of potential injection sites assuming that each injection site comprises, on average, three wellbores. We then adapt the reference resource allocation scenario by constraining the maximum number of injection sites in each region based on historically observed drilling activity.

Finally, we consider a resource allocation order informed by published plans for CO_2_ storage projects in the UK. The first UK carbon storage licensing round in 2023 resulted in 21 licenses awarded to 14 companies across the UK Continental Shelf, with additional licenses from the second round expected in 2027 (see the distribution in Figure 3).^48^ Focusing on storage in saline aquifers, the awarded licenses indicate that near-term deployment is likely to occur initially in the Southern North Sea, where the Endurance project targets the Bunter Sandstone aquifer.^49^ This region also hosts licensed projects such as Viking CCS and Poseidon CCS, which primarily exploit depleted gas fields.^50^ The East Irish Sea is expected to follow via the HyNet North West project, which initially targets depleted hydrocarbon reservoirs but envisions future expansion into saline aquifer storage. In the Central North Sea, the Acorn project is advancing appraisal of the Mey Sandstone and Captain Sandstone aquifers, providing additional large-scale saline storage potential.^51^ The Northern North Sea, despite holding awarded licenses for prospective aquifer storage, is expected to progress later due to its greater distance from shore, more limited cluster connectivity, and comparatively earlier appraisal stage of saline prospects. Accordingly, we consider resource allocation in the sequence: Southern North Sea, East Irish Sea, Central North Sea, and Northern North Sea.

**Figure 3:**
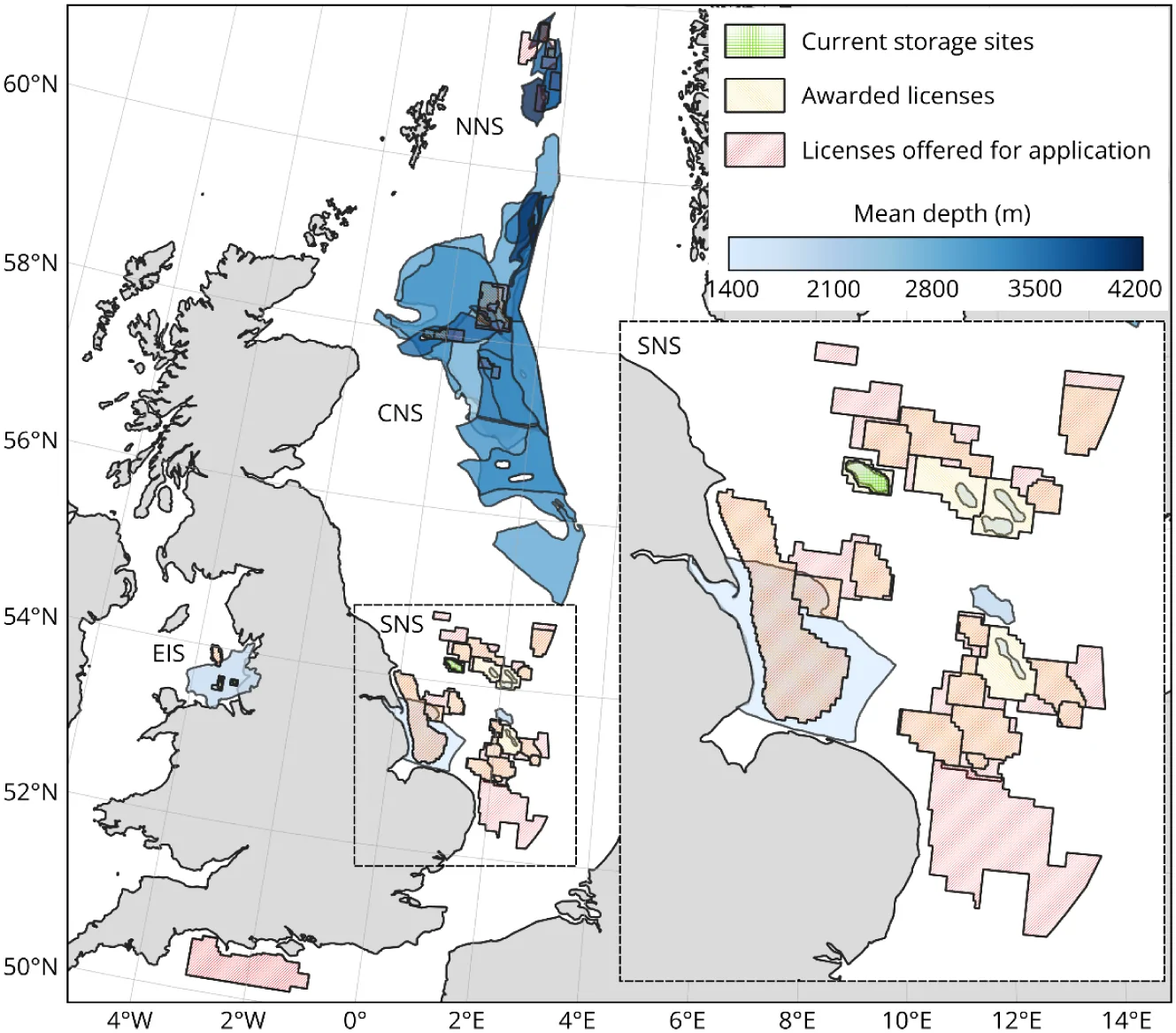
Distribution of the UK offshore (EIS: East Irish Sea, SNS, CNS, and NNS: Southern, Central, and Northern North Sea, respectively) saline aquifers for geological CO_2_ storage together with current storage sites, awarded licenses, and licenses offered for application. The inset shows the distribution of resources and licenses in the southern North Sea. Note that the geographically overlapping saline aquifers in the central and northern North Sea belong to different stratigraphic layers. Storage unit outlines are manually digitized from the CO_2_Stored web viewer and represent approximate extents for illustrative purposes only.

### 2.4 Geologic Storage Units

Data of the storage units are mostly compiled from the CO_2_Stored database.^52,53^ This data set provides information at higher spatial resolution than the comparable data set published by the Geological Service for Europe.^54^ The database contains the parameters required to model reservoir pressure buildup, including storage unit geometry, depth, porosity, permeability, and average reservoir pore pressure, temperature, and salinity. While the database provides estimates of the overburden stress, additional geomechanical parameters required to assess rock failure thresholds, including horizontal in situ stress and rock failure properties, are compiled from published field measurements (explained in detail in Section S2). We also assess the sensitivity of storage resource estimates to uncertainties in reservoir permeability and initial stress conditions. We consider three scenarios in which permeability is reduced by factors of 2, 5, and 10 relative to the reference values from the database while keeping all other parameters unchanged (hereafter denoted k/2, k/5 and k/10, respectively). These scenarios span from moderate uncertainty in the formation-scale permeability to an intentionally conservative end-member represented by k/10, rather than implying that a 10-fold systematic bias in the CO_2_Stored permeability estimates is expected.

Although the initial stress state is arguably set in a conservative way, we define three other scenarios where the initial stress state is changed by increasing the differential stress, i.e., the difference between maximum and minimum principal stresses by 10, 20, and 30% (referred to as 1.1S_d_, 1.2S_d_ and 1.3S_d_, respectively) (Section S2). It should be noted that in contrast to changes in reservoir permeability, which directly control reservoir pressurization in response to injection, changing initial stress alter the safe margin for pressure rise under geomechanical constraints.

We consider CO_2_ storage in saline aquifers, which dominate total storage capacity in the UK. We constrain candidate storage units by considering those with (1) porosity higher than 10%, (2) permeability higher than 1 mD, (3) storage depth greater than 1000 m, where CO_2_ is in supercritical phase to ensure storage efficiency, and (4) total theoretical storage capacity higher than 200 Mt. During this initial screening, the number of potential storage units reduces from 292 saline aquifers initially in the database to 27. All storage units are located offshore, with the majority in the UK stor of the North Sea and only one in the East Irish Sea (Figure 3).

## 3 Results

### 3.1 Growth Model Projections of CO_2_ Storage in the UK

Constraining both logistic and Gompertz models to the UK storage resource of 78 Gt and to midcentury injection targets of 75, 130, and 175 Mt yr^–1^ shows that 2050 lies within an early growth stage rather than near saturation of the resource base (Figure 4). To meet these targets, cumulative storage must increase at average annual rates of 5.8–9.2% between 2030 and the inflection year, and the cumulative storage used by 2050 is low relative to the resource base. The logistic pathways yield 1.15–1.93 Gt resource used by midcentury, while the Gompertz pathways yield 1.25–2.23 Gt. These values correspond to only 1.5–2.9% of the total 78 Gt storage resource.

**Figure 4:**
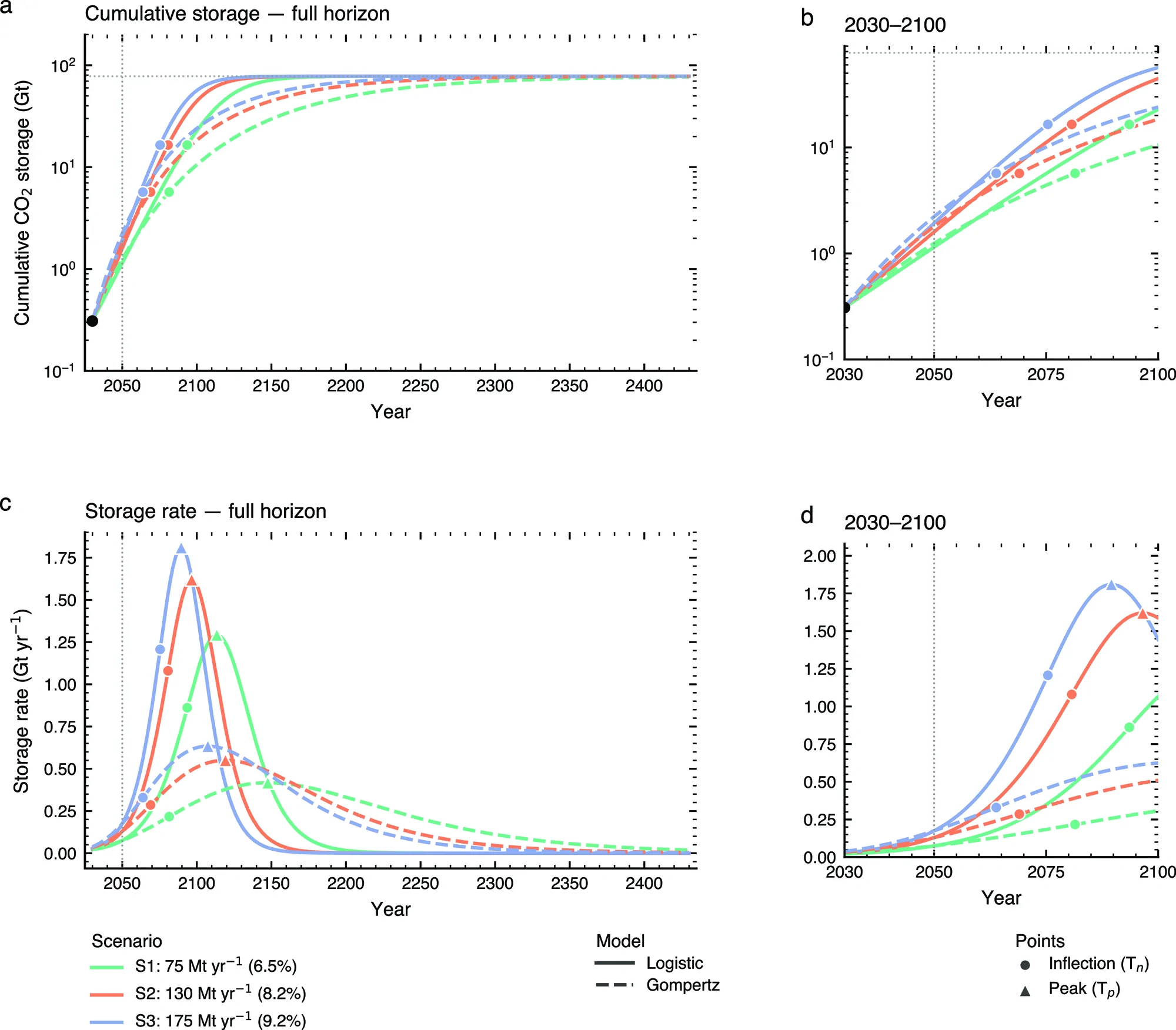
Geological CO_2_ storage scale-up trajectories for the United Kingdom generated using logistic and Gompertz growth models. Panels (a) and (c) show cumulative storage and annual injection rate over the full model horizon (2030–2430), while panels (b) and (d) show the corresponding trajectories for 2030–2100. For each model family, three 2050 injection-rate targets are considered: 75 Mt yr^–1^ (S1), 130 Mt yr^–1^ (S2), and 175 Mt yr^–1^ (S3). Legend labels report the 2050 target rate together with the compound annual growth rate of cumulative storage between 2030 and the inflection year. Vertical dotted lines mark 2050, and the horizontal dotted line in panels (a) and (b) denotes the assumed 78 Gt geological storage capacity.

This can be understood more clearly by considering the positions of the inflection year and the peak year in the growth models relative to the geological resource base. These points are fixed by the geometry of each model family and do not depend on the chosen 2050 target. For the logistic model, the inflection occurs at around 21% total geological capacity, equivalent to 16.5 Gt, and the peak rate coincides with the consumption of exactly half the resource at 39.0 Gt. For the Gompertz model, the corresponding values are much earlier in cumulative resource use, with 5.7 Gt for the inflection year, after storing roughly one-third of the cumulative mass required to reach the inflection year in the logistic model, and 28.7 Gt for the peak year. In other words, the Gompertz trajectory reaches both inflection and peak after a substantially smaller fraction of the total resource has been used. This difference arises from the intrinsic asymmetry of the Gompertz form and is the main reason why the two model families diverge so strongly after 2050.

These geometric anchors also translate into different temporal and intensity profiles for the scaleup. In the logistic family, the interval between inflection and peak is short, only 14–20 years. In the Gompertz family, the same interval extends to 44–66 years, roughly 3 times longer. This produces a marked contrast in peak-rate requirements. Logistic pathways reach peak injection rates of 1.29–1.81 Gt yr^–1^, an order of magnitude larger than the 2050 target. Gompertz pathways peak at only 0.42–0.63 Gt yr^–1^, about 3.6–5.6 times the corresponding 2050 target. For the most ambitious 175 Mt yr^–1^ scenario, the logistic peak reaches 1.81 Gt yr^–1^, compared with 0.63 Gt yr^–1^ for the Gompertz case, for the same 2050 rate target and converge to the same ultimate storage resource. The choice of growth model, therefore, has major consequences for the peak injection rate, and this in turn has an impact on the evaluation of the dynamic geophysical limits. A later inflection, as in the logistic case, concentrates the burden of scaleup into a shorter period and drives much higher peak injection demand. An earlier inflection, as in the Gompertz case, distributes that burden over a longer time window and lowers the required peak rate.

The significance of inflection year for near-term planning is most evident when comparing its timing relative to 2050, where we have imposed the rate constraint. Across all six scenarios, the inflection year falls between 2064 and 2094, placing 2050 some 14–44 years before the end of the strongly accelerating phase. By 2050, only 7–12% of the cumulative storage required to reach the inflection year has been delivered in the logistic cases, compared with 22–39% in the Gompertz cases.

Thus, midcentury policy targets do not probe the ultimate capacity of the UK subsurface. Rather, they test how rapidly the sector can progress through the early part of a much longer scaleup pathway. This point is important for the analysis that follows, because the geophysical challenge is not whether the UK can accommodate storage by 2050, but whether the much larger postinflection injection rates implied by different growth trajectories remain compatible with subsurface injectivity limits.

### 3.2 Injectivity-Limited Resources of the UK Offshore

The total storage capacity provided by the 27 storage units selected in this study approaches 79 Gt over a 100-year injection period, corresponding to a regional injection rate of 790 Mt yr^–1^. Detailed injectivity calculations, optimization of injection-site configurations, and storage capacity estimates of all units are detailed in Section S3 of the Supporting Information. Nearly 630 injection sites are required to achieve this rate (Table S1). The largest contributions come from Mey 5 and Maureen 2 with 28.5 and 14 Gt storage capacities in the central and northern North Sea, respectively (Figure 5). These large capacities reflect their considerable areal extent, thickness, and permeability, favorable stress conditions and open boundary flow regimes. Indeed, while units with open boundary flow conditions constitute only one-quarter of the considered storage units, they provide more nearly 70% of the total storage capacity. Contributions from confined units are dominated by Cormorant 009 18 in the northern North Sea and Collyhurst SS Fm 1 in the East Irish Sea, which together offer an additional ∼12 Gt of cumulative storage resource.

**Figure 5:**
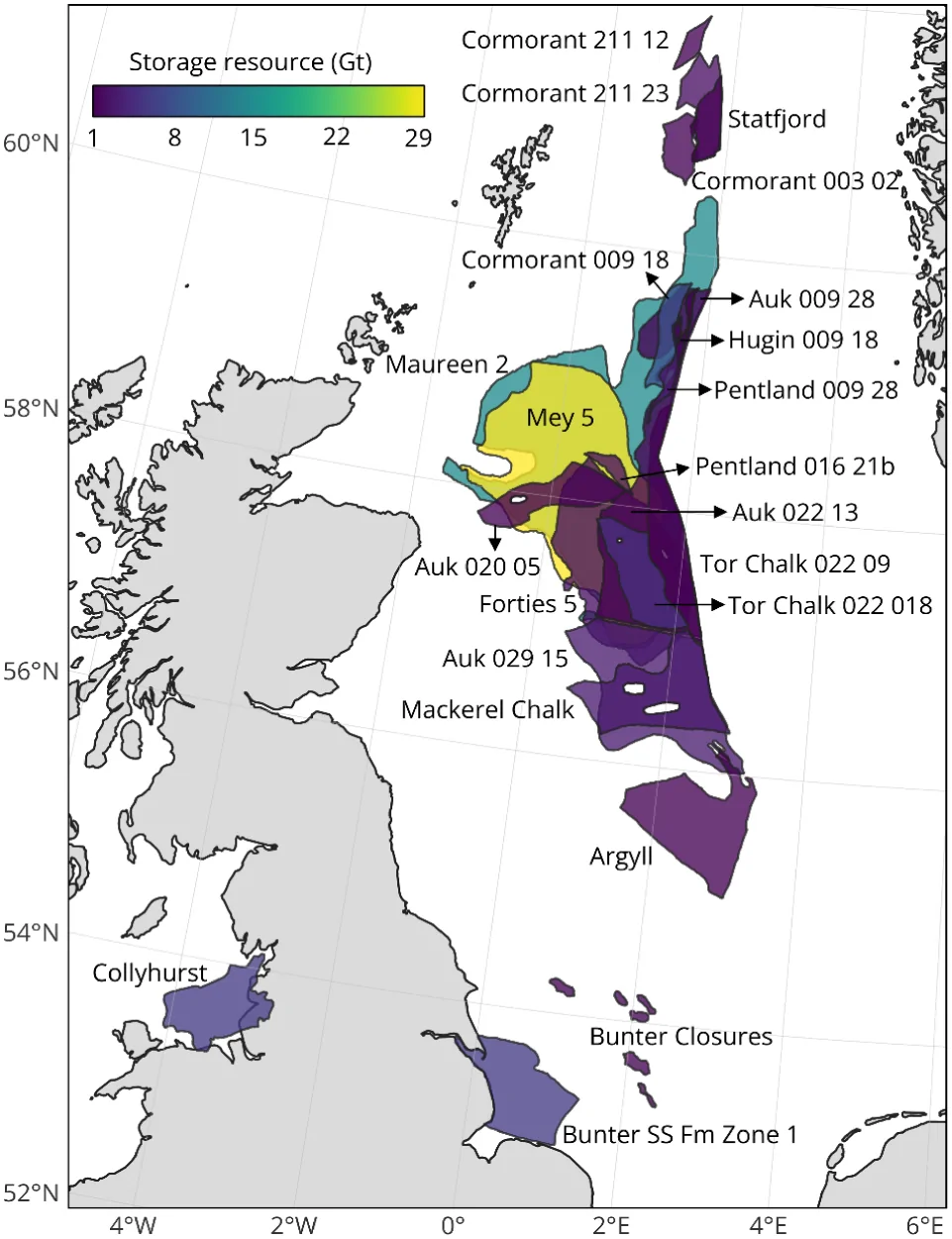
Spatial distribution of CO_2_ storage resources in the UK offshore saline aquifer calculated for 100 years of continuous injection at constant rates. The digits following the storage unit names are numerical labels used in the CO_2_Stored database to distinguish individual storage units.

In contrast, several storage units cannot withstand pressure buildup associated with injection rates above the minimum threshold of 1 Mt yr^–1^ even through one injection site. Their effective storage capacities therefore fall below the minimum viable level and are excluded from the analysis here (capacities denoted by “-” in Table S1). These units include several small Bunter Closures in the southern North Sea. Overall, the southern, central, and northern North Sea and East Irish Sea account for approximately 7%, 56%, 31%, and 6%, respectively, of the total estimated UK offshore storage resources (Figure 5). The obtained results underscore the substantial storage potential of the central and northern North Sea regions and highlight their critical role in meeting national CO_2_ storage scaleup trajectories.

### 3.3 Injectivity Limits to CO_2_ Storage Scale-Up

#### 3.3.1 Logistic Growth Trajectories

The resource allocation analysis shows that the logistic growth trajectories targeting CO_2_ storage rates of 75, 130, and 175 Mt yr^–1^ by 2050 and a cumulative storage amount of 78 Gt cannot be fully accommodated within the assessed UK offshore storage units (Figure 6). Although sufficient storage resources exist to accommodate these cumulative storage targets (Section 3.1), the peak injection rates implied by the growth models exceed the aggregate injectivity of the available storage units. This observation is independent of the resource allocation sequence, although the allocation strategy that prioritizes smaller-capacity units accommodates slightly lower peak injection rates and cumulative storage amounts than the strategy prioritizing larger units (compare Figure 6a and b). The observed trend indicates that the limiting factor for these trajectories is the achievable injection rate rather than the total storage resource.

**Figure 6:**
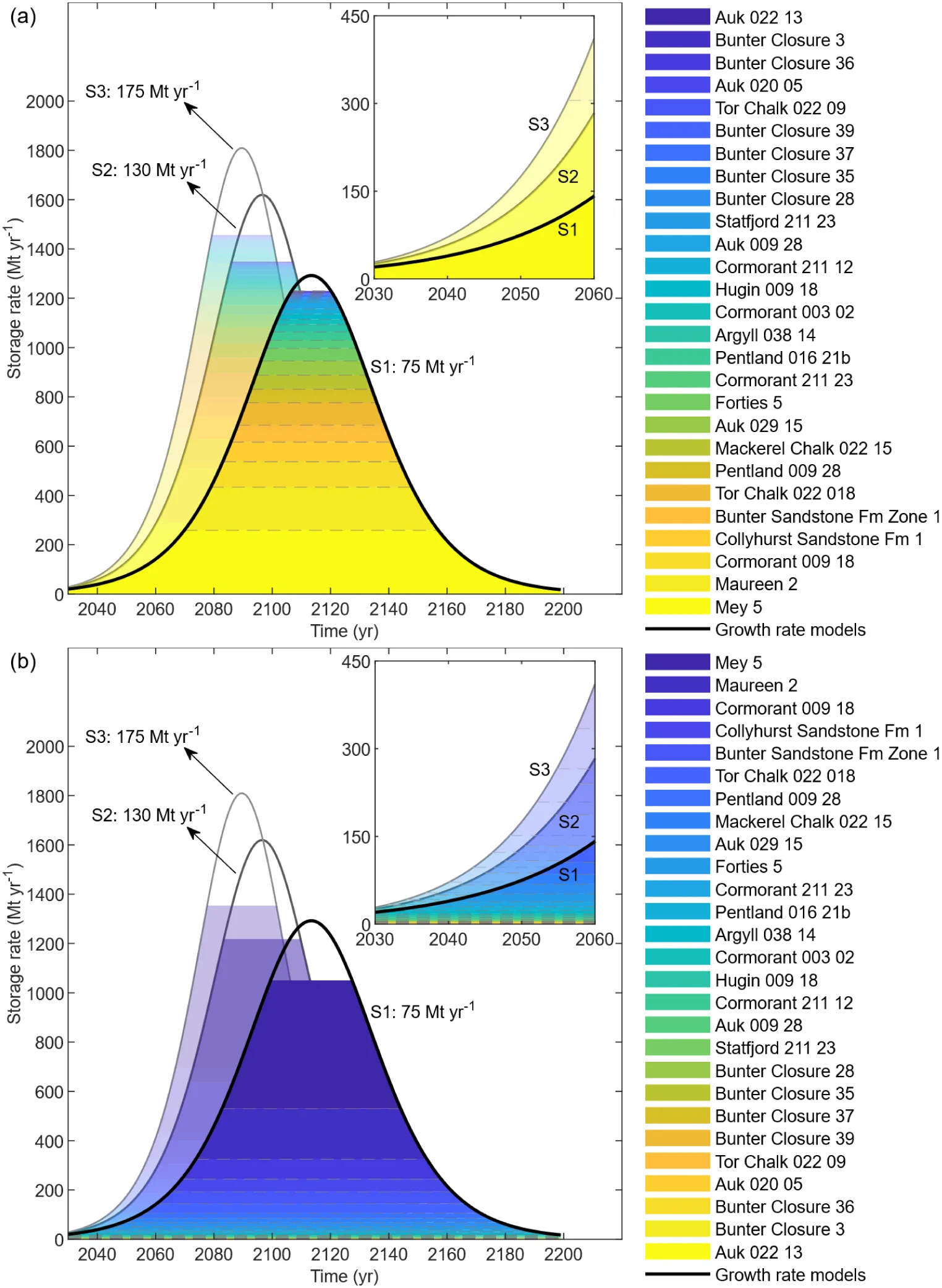
Screening allocation of resources in (a) descending and (b) ascending order of capacity, under reservoir injectivity constraints, for three growth rate models targeting CO_2_ storage rates of 75 Mt yr^–1^ (S1), 130 Mt yr^–1^ (S2), and 175 Mt yr^–1^ (S3) by 2050 and a cumulative storage resource of 78 Gt. Insets show zoomed views of the plots during the early stages of injection.

Having now identified that the reference logistic growth trajectories cannot be achieved due to dynamic geophysical limits (reservoir pressurization), the modeling framework updates the growth trajectory to a path that can be achieved without overpressuring the system. Lowering the peak injection rate in a logistic curve while maintaining the starting point and 2050 rate targets is carried out by reducing the total static storage resource. A growth trajectory with a 2050 storage rate of 175 Mt yr^–1^ (the most aggressive target 2050 rate considered in this study), with a corresponding peak injection rate of approximately 1.6 Gt yr^–1^ results in a cumulative storage amount of 69 Gt (Figure 7).

**Figure 7:**
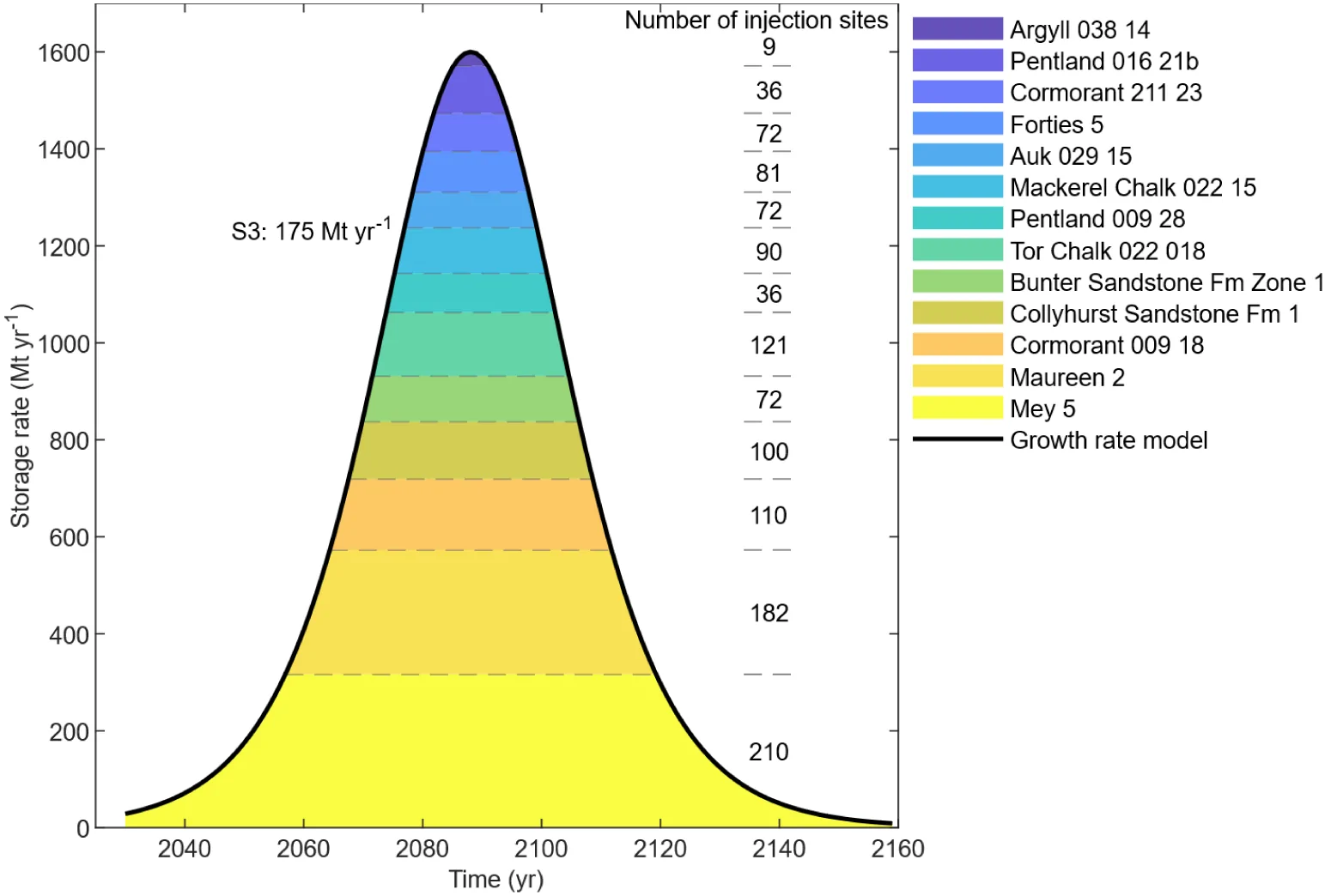
Resource allocation under the growth rate model targeting a 2050 storage rate of 175 Mt yr^–1^ and a cumulative storage amount of 69 Gt, for the scenario prioritizing the largest units.

An important outcome of this modeling framework is the ability to identify the static storage resource base that can be used along feasible growth trajectories, under dynamic geophysical constraints. Identifying a resource base of 69 Gt, for example, suggests that there is some risk that the 78 Gt represents an overestimate of the storage resource base relative to what can be achieved under growth and geophysical constraints. However, as will also be discussed below, ultimately the 78 Gt estimate falls well within the resulting range of outcomes emerging from the exploration of uncertainty in parameters.

The second end-member allocation scenario, prioritizing the smallest storage units, still allows this scaleup trajectory without meeting geophysical limitations, although this allocation scenario would be more resource-intensive (Figure S8). Twenty storage units would be needed to accommodate the scaleup to 2050, and all of the storage units would be needed to accommodate trajectory across the entirety of the logistic trajectory. The reason for the observed differences between the two resource allocation strategies is mainly 2-fold. First, several small units considered for long-duration injection can not tolerate large injection rates and therefore contribute only marginally to the cumulative storage amount. Second, assigning large-capacity units with open flow boundary regimes like Mey 5 and Maureen 2 to elongated injections is advantageous because sufficient time is provided for part of the excess pressure to dissipate at boundaries, slightly expanding the storage capacity.

Analysis of 100 random allocation scenarios shows that the majority of scenarios are capable of addressing the Q_2050_ = 175 Mt yr^–1^ target and a cumulative storage amount of 69 Gt (Figure 8). Only three scenarios marginally fail to meet this target. The number and specific sequences of failed allocations may slightly vary between sets of random realizations, with no systematic pattern identified. This behavior is because the maximum injection rate that can be sustained by each storage unit depends on the injection duration, with additional nonlinearities introduced by constraints on per-site injection rates and intersite distances. Therefore, each unit can be considered to have an optimal contribution to the growth trajectory for a particular duration. Depending on its position in the allocation sequence, a unit may operate away from this optimum, slightly reducing its contribution to addressing the trajectory. The accumulation of these small reductions across multiple units ultimately appear as a small gap at the end of the allocation, preventing the full trajectory from being accommodated. The required number of geological units also varies with the allocation sequence but remains within the range defined by the two allocation strategies described above. This trend supports the interpretation that these resource allocation strategies represent end-member cases. Consequently, any realistic allocation pathway lying between these extremes would be sufficient to meet the projected scaleup trajectory.

**Figure 8:**
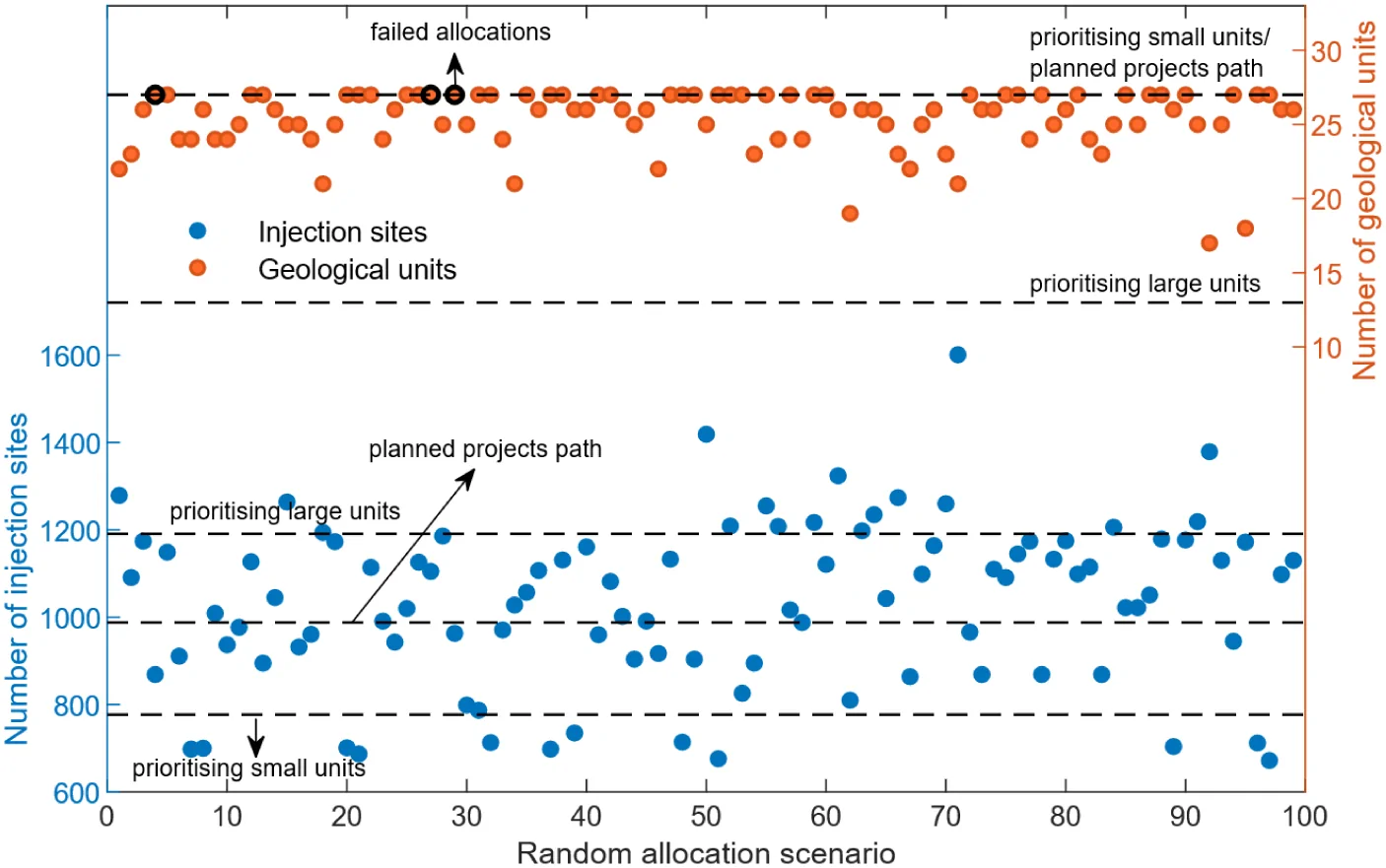
Number of injection sites (left axis) and geological units (right axis) across random allocation scenarios. Horizontal dashed lines indicate key allocation strategies, including prioritization of large units, prioritization of small units, and the scenario consistent with the planned projects pathway. Failed allocation sequences are highlighted by black outlines.

A plausible allocation sequence consistent with currently planned CO_2_ storage developments prioritizes the southern North Sea, the East Irish Sea, the central North Sea and the northern North Sea. The results indicate that this allocation pathway can also accommodate growth trajectories with a storage resource base of 69 Gt (Figure S9), further demonstrating that the conclusion is largely independent of the specific order in which storage resources are developed. Furthermore, the demonstrated ability to allocate resources for the most aggressive growth rate model achieving 175 Mt yr^–1^ injection rate by 2050 means that any other considered trajectory with a smaller 2050 target injection rate but similar cumulative storage mass can be safely addressed.

The resource allocation analysis highlights the extensive infrastructure requirements associated with large-scale CO_2_ storage deployment. Approximately 210 injection sites within the Mey 5 unit are needed once the full injection rate of 300 Mt yr^–1^ is attained. However, achieving the peak injection rates projected by logistic growth models necessitates a much larger number of injection sites for optimized use of the storage resources. At its peak rate of 1.6 Gt yr^–1^, nearly 1,200 injection sites are needed under the reference resource allocation approach, i.e., prioritizing larger units (Figure 7). This high demand implies the combined effects of long injection durations, the imposed technological upper limit of 20 Mt yr^–1^ per site, and local injectivity constraints associated with unfavorable reservoir properties. The latter factor is exemplified by the Tor Chalk 022 018 unit, where a reservoir permeability of only 5 mD entails use of 121 injection sites to maximize its contribution to addressing the scaleup trajectories (Figure 7).

The total number of injection sites across 100 random resource allocation scenarios ranges from approximately 700 to nearly 1,600 (Figure 8). The first allocation scenario lies toward the upper bound of this range, whereas the conservative allocation strategy, i.e., prioritizing small-capacity units, with 780 injection sites, and the sequence consistent with currently planned projects, with 990 injection sites, lie close to the lower bound and the mean of the distribution, respectively (Figure 8). This variability occurs because the number of sites deployed within each storage unit differs substantially between scenarios, primarily as a function of injection duration. In particular, large-capacity units assigned to long injection periods under the first allocation strategy require a larger number of injection sites operating at relatively low rates to maximize storage utilization. In contrast, when these units are allocated later in the sequence and over shorter injection periods in the second allocation strategy, fewer sites operating at higher injection rates are sufficient to achieve the required storage contribution. As such, the framework optimizes injection grid design to maximize total injectivity and cumulative storage capacity, rather than to minimize the number of injection sites, and the resulting site counts emerge as a secondary outcome of this optimization.

Reducing the maximum number of injection sites in each geological unit, based on the historical number of hydrocarbon wells drilled in the corresponding region, imposes an additional constraint on storage deployment. This decreases the maximum attainable peak injection rate to approximately 1.5 Gt yr^–1^ and the cumulative storage amount to 64 Gt (Figure S10). The Mey 5 and Maureen 2 units are exceptions, as their contribution to the growth trajectories remains unaffected owing to the large number of existing wellbores (Figure S10). In contrast, Tor Chalk 022 018 is no longer among the major contributing storage units because of the limited historical drilling activity in this region combined with its relatively low injectivity. In this setting, nearly 880 injection sites are still sufficient to achieve the target storage rate of 175 Mt yr^–1^ by 2050 with the lowered associated storage resource base of 64 Gt.

#### 3.3.2 Gompertz Growth Trajectories

In contrast to the logistic growth models, the Gompertz growth model fully accommodates the scaleup pathway targeting a storage rate of 175 Mt yr^–1^ by 2050 and a cumulative storage amount of 78 Gt (Figure 9). This difference arises from the slower growth and decline phases of the Gompertz model, which reduce the peak injection rate to approximately 640 Mt yr^–1^. The combination of longer injection durations and lower peak rates reduces pressure interference between injection sites and allows for more sustainable and efficient utilization of storage resources. In particular, the elongated injection strategy substantially increases the contribution of Mey 5 (∼41 Gt) relative to the logistic growth trajectories (∼27 Gt) (compare Figures 9 and 7), as its high injectivity and open boundary conditions allow pressure dissipation over long injection durations. Consequently, a larger fraction of the total storage resource can be accommodated within a small number of highly favorable storage units.

**Figure 9:**
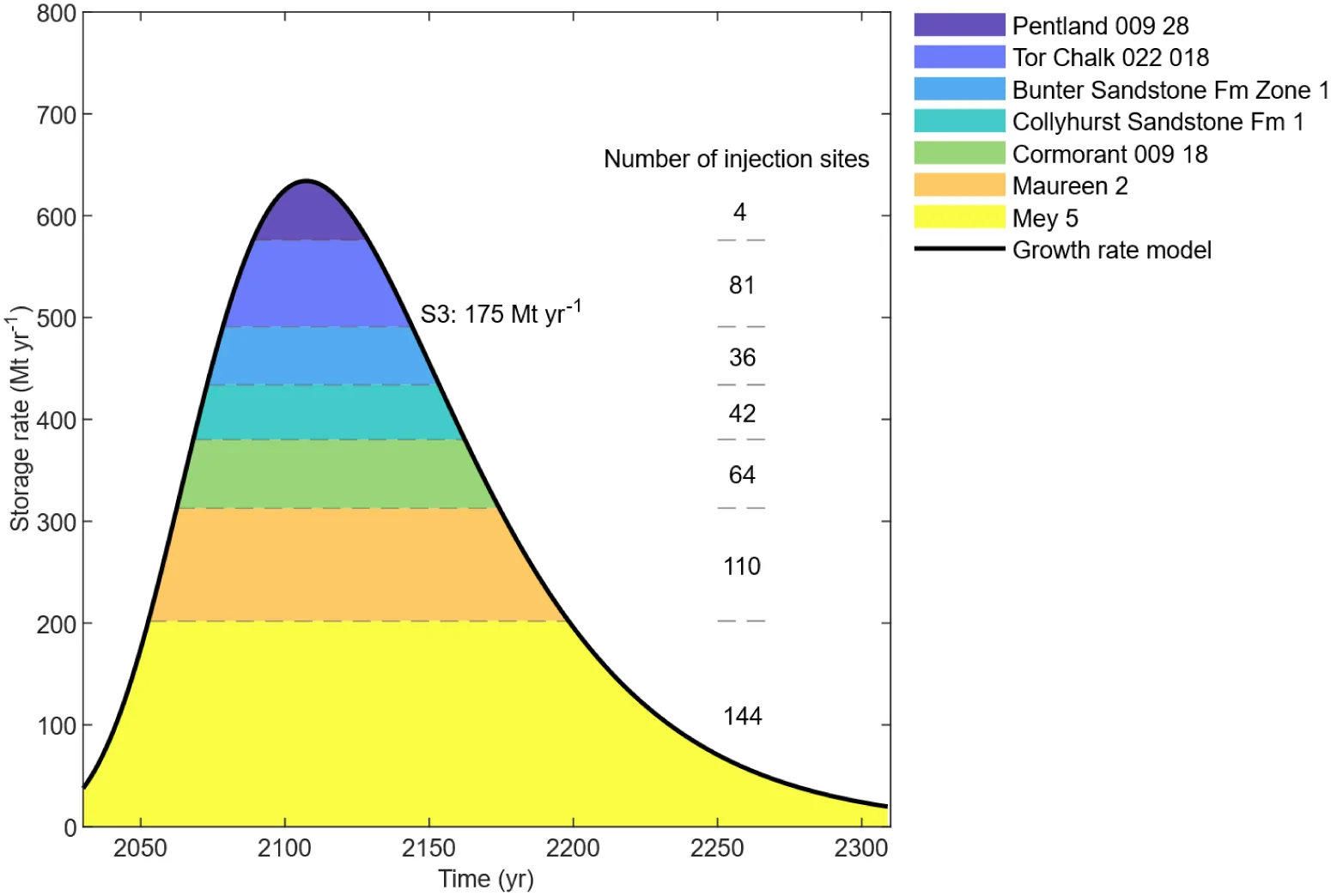
Resource allocation for the Gompertz model targeting a CO_2_ storage rate of 175 Mt yr^–1^ by 2050 and a cumulative storage mass of 78 Gt. Note that the scales are different from the plots for the growth rate model.

The reduced peak injection rate without substantial loss of per-site injectivity is translated into a smaller number of required injection sites under Gompertz trajectories. While the logistic growth trajectory with a storage resource of *C* = 69 Gt requires approximately 1200 injection sites under the reference allocation strategy (Figure 7), the Gompertz trajectory with the storage resource *C* = 78 Gt can be achieved using only 477 injection sites (Figure 9). This reduction demonstrates the strong dependence of storage deployment requirements on the assumed growth trajectory and corresponding temporal evolution of injection rates.

#### 3.3.3 Impact of Uncertainties in Reservoir Permeability and Stress State

The obtained results so far are based on reservoir pressurization models parametrized using the most likely hydraulic properties reported in the CO_2_Stored database and reported regional stress measurements. When uncertainty is introduced in reservoir permeability, the 2050 targets can still be met, but the long-term scaleup trajectories discussed so far become increasingly difficult to sustain. As permeability decreases, pressure limitations become progressively more restrictive, reducing both the peak injection rates and cumulative storage amounts that can be attained. This effect is evident for the Gompertz trajectories targeting a 2050 storage rate of 175 Mt yr^–1^ (Figure S11). While this trajectory can be achieved with a total storage resource of 78 Gt under the reference permeability values, a 10-fold reduction in permeability precludes achieving the required peak injection rates due to pressure constraints, reducing the achievable storage resource base from 78 to 41 Gt. In all cases of reduced permeability by factors from 2 to 10, the achievable storage resource base is reduced from 78 Gt (Figure 10).

**Figure 10:**
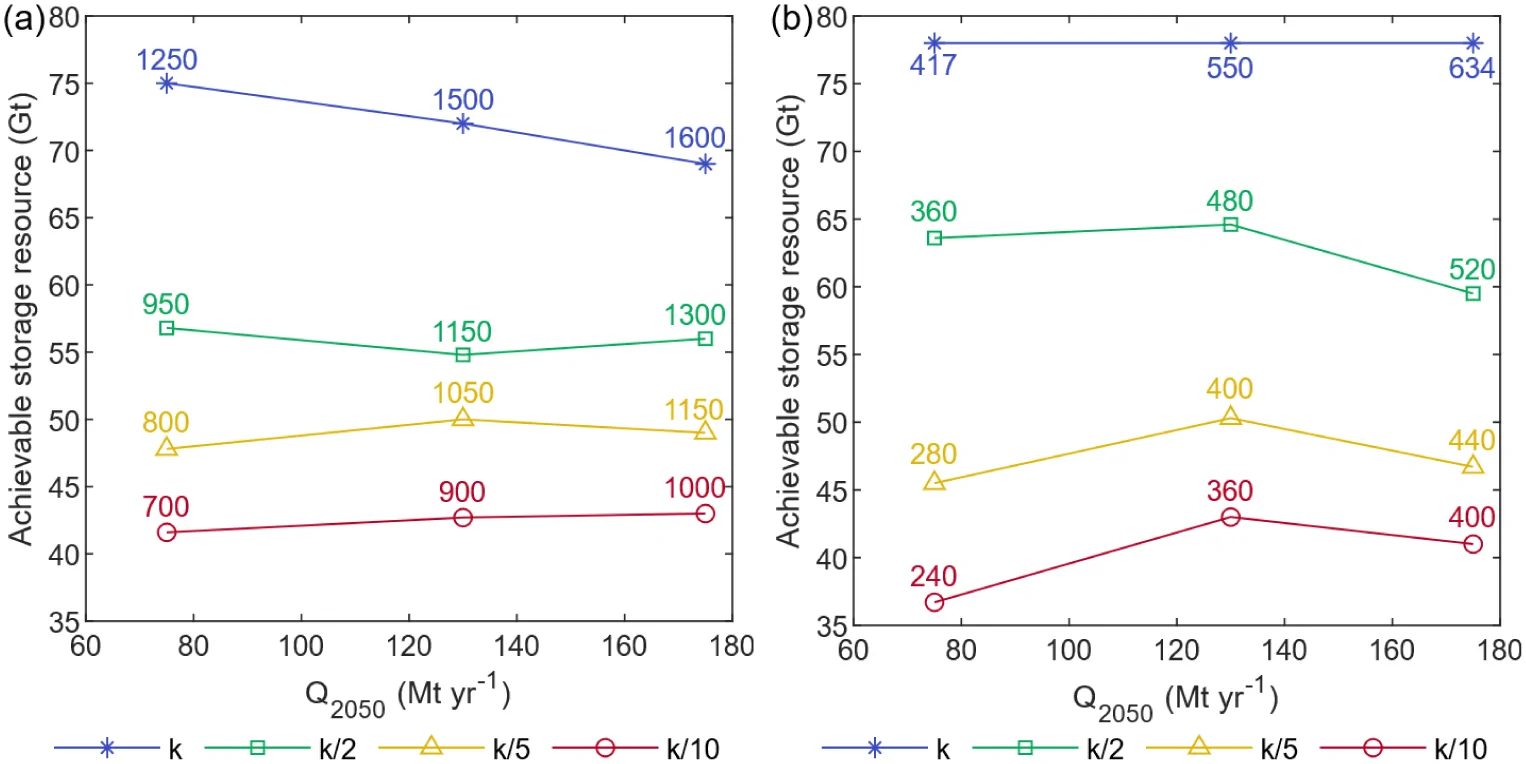
Estimated achievable storage resource for (a) logistic and (b) Gompertz growth trajectories targeting 75, 130, and 175 Mt yr^–1^ by 2050, considering uncertainties in reservoir permeability: most likely permeability values from the CO_2_Stored databased (*k*) and reduced by factors of 2 (*k*/2), 5 (*k*/5), and 10 (*k*/10). The corresponding peak injection rates (Mt yr^–1^) for each scenario are shown as labels.

As previously described, imposing lower peak injection rates while maintaining a storage rate in 2050 is equivalent to reducing the storage resource base in the growth models. We thus use this modeling framework to identify the sensitivity of the achievable storage resource base to uncertainty. We generate alternative trajectories by lowering the maximum allowable peak injection rate in steps of 50 Mt yr^–1^ and 40 Mt yr^–1^ relative to the reference cases for logistic and Gompertz trajectories, respectively, while maintaining *Q*_2050_. The resulting trends show a nonlinear decrease in the achievable storage resource base with decreasing permeability (Figure 10). For logistic trajectories, even a 2-fold reduction in permeability (*k*/2) leads to a marked reduction in both peak injection rates and achievable storage resource (Figure 10a). A 10-fold permeability reduction (*k*/10) reduces the attainable peak injection rates by ∼600 Mt yr^–1^ and the cumulative storage amount by ∼30 Gt relative to the reference case. Within Gompertz trajectories, achievable injection rates decrease more gradually with permeability reduction owing to their lower peak injection rates and more uniformly across the injection duration (Figure 10b). As a result, the reduction in cumulative storage remains comparable to, and in some cases exceeds, that observed for logistic trajectories. Thus, although Gompertz trajectories are less constrained by peak-rate reductions, their ultimate cumulative storage remain sensitive to permeability uncertainty. Yet, substantial deployment including all *Q*_2050_ targets remain achievable even under severe reductions in permeability.

Uncertainty in the initial state of stress also has substantial impact on the achievable storage resource. In particular, logistic growth trajectories respond in a manner similar to reductions in reservoir permeability, with increasing differential stress substantially reducing the achievable storage resource (Figure S12a). However, Gompertz trajectories are less sensitive to changes in the initial stress state. The achievable storage resource remains unchanged when the differential stress is increased by a factor of 1.1 and is consistently larger than that of logistic growth trajectories under larger stress perturbations (Figure S12b). This trend can be attributed to lower peak injection rates achieved in Gompertz trajectories, which maintain a relatively large margin from reservoir failure threshold. Consequently, moderate changes in the initial stress state have limited influence until this geomechanical margin is sufficiently reduced.

## 4 Discussion

Our analysis shows that CO_2_ storage scaleup trajectories considered for the UK can be accommodated without exceeding critical geophysical limits. Even under severe reductions in reservoir permeability and increases in the initial differential stress, all UK government midcentury deployment targets, including achieving storage rates of 175 Mt yr^–1^, remain feasible. This conclusion is derived despite the conservative exclusion of a number of aquifers during initial screening because of their less favorable storage characteristics. However, reservoir pressurization imposes meaningful constraints on the pace and ultimate scale of storage deployment. Thus, while reservoir pressurization is unlikely to constrain the achievement of national-scale midcentury targets, it may limit the long-term growth and cumulative storage that can ultimately be attained. Instead, the primary limiting factor emerges from the achievable pace of industrial expansion to meet climate change mitigation roadmaps, although determining the feasibility of such expansion requires further assessment of the underlying techno-economic constraints.

The ability of UK offshore saline aquifers to accommodate aggressive CO_2_ storage growth trajectories is underpinned by the large dynamic storage resources estimated in this study, which approach 79 Gt when assuming injection at a constant, sustainable rate over a century. This estimate exceeds the pressure-limited storage capacity of approximately 22 Gt derived by de Jonge-Anderson et al. (2026),^38^ is moderately higher than the ∼71 Gt estimated by Smith et al. (2024),^55^ and remains below the ∼140 Gt reported by De Simone and Krevor (2021).^36^ The discrepancies likely reflect differences in geological representation and modeling assumptions. de Jonge-Anderson et al. (2026)^38^ and Smith et al. (2024)^55^ employed the spatially coarse-resolution GSEU^54^ and global sedimentary^56^ databases, both excluding the overlapping storage units and assuming closed boundary flow conditions. In contrast, the more detailed representation of geological units adopted here suggests open-flow boundary conditions for a number of aquifers, enabling lateral pressure dissipation and increasing accessible pore space. Additionally, the use of refined geophysical parameters, particularly field-constrained estimates of in situ stress and effective rock compressibility further contributes to the larger storage resources estimates. Although De Simone and Krevor (2021)^36^ employed a similar geological database (CO_2_Stored database), the lower estimates obtained here is primarily because of the use of region-specific geomechanical constraints, the application of a minimum technical injection-rate threshold for site development, and improvements to CO2BLOCK-GROWTH for closed-boundary pressure buildup calculations.

We show that reaching Gt-scale injection rates outlined by growth rate models requires up to ∼1,600 injection sites (Figure 8), depending on the scaleup trajectory and resource allocation strategy. This corresponds to approximately 4,800 injection wellbores assuming three wells per site. This scale of deployment is consistent with previous regional and global assessments. At the UK scale, de Jonge-Anderson et al. (2026)^38^ highlight the need for nearly 800 injection wellbores for storage scaleup to 300 Mt yr^–1^ by 2050. Ringrose and Meckel (2019)^25^ also suggest that achieving the IEA^57^ projected ∼7 Gt yr^–1^ of CO_2_ storage by 2050 would require around 10,000 injection wells worldwide. Although these numbers may appear large, they are not unprecedented. Over six decades of hydrocarbon exploration and production, the UK continental shelf has cumulatively hosted more than 13,000 offshore wellbores with peak drilling activity exceeding 400 wells per year.^47^ Nearly 6,000 wells have penetrated candidate UK offshore storage aquifers, still exceeding the number required to meet the projected growth trajectories. This level of historical subsurface development in terms of offshore engineering, logistic and regulatory capabilities needed to deliver high-density well networks renders achieving national CO_2_ storage scaleup targets technically plausible.

A further technoeconomic consideration arises from the fact that the storage units providing the largest contributions, particularly in the central and northern North Sea, involve deep formations, often exceeding 2.5 km depth. These depths may offer enhanced CO_2_ containment underground by (1) promoting residual and dissolution trapping mechanisms, particularly, in thick and dipping formations,^58^ (2) enhancing the likelihood of forming secondary CO_2_ accumulations in multilayered sedimentary settings,^59,60^ and (3) increasing mechanical stability by elevating tensile failure limits of the caprock.^36^ They also reduce upward pressure propagation toward shallow groundwater.^61^ However, well construction and operational costs may significantly increase with depth.^62^ Consequently, despite excellent injectivity and capacity offered by these deep units, their development entails sustained investment and supportive policy frameworks. Therefore, the UK’s largest storage resources are technically available but economically demanding.

The present analysis is restricted to offshore saline aquifers because they account for the majority of the estimated UK storage resources,^63,64^ motivating our focus on these aquifers for assessing multidecadal CO_2_ storage scaleup. However, current UK storage licenses also include depleted oil and gas fields,^44,48^ which are not represented in our analysis. Depleted hydrocarbon fields may be particularly attractive for near- to midterm deployment because of their extensive subsurface characterization, potential for infrastructure reuse, and reduced pore pressure, which can provide additional pressure margin and potentially enhance CO_2_ injectivity and storage capacity.^65^ While achieving near-term targets such as the 2050 storage rate is not geophysically constrained, including depleted hydrocarbon fields could facilitate more rapid and cost-effective development during the early stages of UK CO_2_ storage scaleup.

Our resource-allocation framework assumes that all injection sites assigned to a given unit are available from the beginning of its operational period. In reality, CO_2_ storage development will follow a sequential progress, beginning with a small number of hubs and expanding over time as the infrastructure, permitting, and transport networks become mature.^50^ This phasing should still not compromise reservoir injectivity as injection rates remain far below critical thresholds. However, our analysis indicates that the largest regional storage rates are achieved through dense grids of injectors operating at relatively low per-site rates. While a smaller number of high-rate injectors can deliver comparable cumulative rates, they may delay the ramp-up of national storage rates and pose difficulty in reaching more ambitious midcentury targets. The initial gradual rise in storage rates can be compensated for by elongating the high-injection period, which is also compatible with geophysical limits, as it allows for more sustainable use of the subsurface resources. Such scaleup profiles may represent more realistic trajectories for achieving ambitious UK-wide CO_2_ storage goals.

The use of pressure superposition overestimates injection-induced overpressure,^46^ making our storage resource estimates conservative. However, the extent to which more realistic pressure estimates would expand the storage scaleup depends on the complex nonlinear relationships among pressure buildup, storage rate evolution, site configuration, and storage capacity, and remains to be quantified. An additional conservative assumption arises from the use of regular, uniform site patterns within each storage unit. In practice, well placement can be optimized to address geological heterogeneities and minimize reservoir pressurization.^66^ Basin-scale pressure management strategies, such as brine extraction or coordinated operation across neighboring units, could further reduce pressure buildup and unlock additional storage capacity.^67,68^ This suggests that our estimates of injectivity and storage capacity represent lower bounds on what could ultimately be achieved under optimized operations. Such site- and field-level development strategies warrant detailed subsurface characterization and high-resolution, multiphysics simulations that acknowledge subsurface structural and physical complexities, which fall beyond our screening-level study.

Despite remaining geophysical uncertainties and methodological simplifications, the conducted sensitivity analyses suggest that the key conclusions of this study remain robust. Substantial UK CO_2_ storage scaleup trajectories remain feasible within geophysical constraints, suggesting that large-scale deployment will primarily depend on the ability to sustain the required industrial, infrastructural, and investment growth. This indicates that the UK’s offshore storage resources are sufficient to support large-scale decarbonization, provided that policy, investment, and industrial capacity expand at a pace consistent with national climate commitments. More broadly, the framework developed in this study is transferable to other national and regional contexts, thereby supporting the assessment of CO_2_ scaleup feasibility globally. The incorporation of this framework into integrated assessment models would enable a more realistic representation of the timing, scale, and physical limits of subsurface storage in long-term decarbonization pathways.

## Supplementary Material



## Data Availability

The scripts for the CO2BLOCK-GROWTH tool, along with the input files used for resource estimates and allocations in the UK CO_2_ storage scale-up study, are publicly available at https://github.com/ImperialCollegeLondon/CO2BLOCK/.

## Notes

Additional details of the dynamic storage resource assessment and geophysical model parametrization; supplementary analyses of reservoir injectivity, resource allocation, and geological uncertainty, including supplementary figures and table (PDF)
