# Supplementary material for: Coupled Geophysical and Technoeconomic Industrial Growth Constraints on Geological Carbon Storage Scale-Up: Application to UK Offshore Resources
Source: Environ Sci Technol. 2026 Nov 2;60(44):31463–78. doi: 10.1021/acs.est.6c10305 (PMC13757958; doi:10.1021/acs.est.6c10305)
Supplement: Supplementary file 1 [file es6c10305_si_001.pdf]

Supporting Information for

Coupled geophysical and technoeconomic industrial growth constraints on geological carbon storage scaleup: application to UK offshore resources

Iman R. Kivi[1,2], Xiaowei Gao[2], Samuel Krevor[2]

[1]Global Change Research Group, Mediterranean Institute for Advanced Studies, University of the Balearic Islands, 07190 Esporles, Balearic Islands, Spain

[2]Department of Earth Science and Engineering, Imperial College London, London SW7 2AZ, United Kingdom

S1. Dynamic storage resource assessment

In order to assess subsurface storage resources constrained by reservoir pressurisation and understand their control on achieving geologic CO_2 storage scaleup trajectories represented by growth models, we have adapted the analytical tool CO2BLOCK (Fig. S1). The tool was originally developed for simplified screening of subsurface CO_2 storage resources (De Simone et al., 2019; Kivi et al., 2025). It employs analytical solutions for two-phase flow of CO_2 and brine in saline aquifers under open and closed boundary flow conditions to calculate the spatial and temporal evolution of pore pressure around injection sites. By superimposing pressure fields of multiple injection sites, distributed in specific rectangular patterns and at varying distances across each of the storage regions, CO2BLOCK enables rapid evaluation and optimisation of basin-scale pressurisation under diverse injection scenarios. The analytical model for basin-scale pressure buildup estimates is explained in detail below.

Considering pressure buildup as the primary mechanism governing mechanical stability of subsurface structures, CO2BLOCK compares the estimated pressure evolution with rock failure criteria to identify the maximum sustainable injection rates. This modelling framework allows for an estimate of the storage capacity that can be safely achieved while maintaining the mechanical integrity of the storage complex. In particular, CO2BLOCK accounts for constraints imposed by caprock fracturing threshold and shear reactivation of pre-existing faults, which could compromise permanent containment of CO_2 deep underground or induce earthquakes.

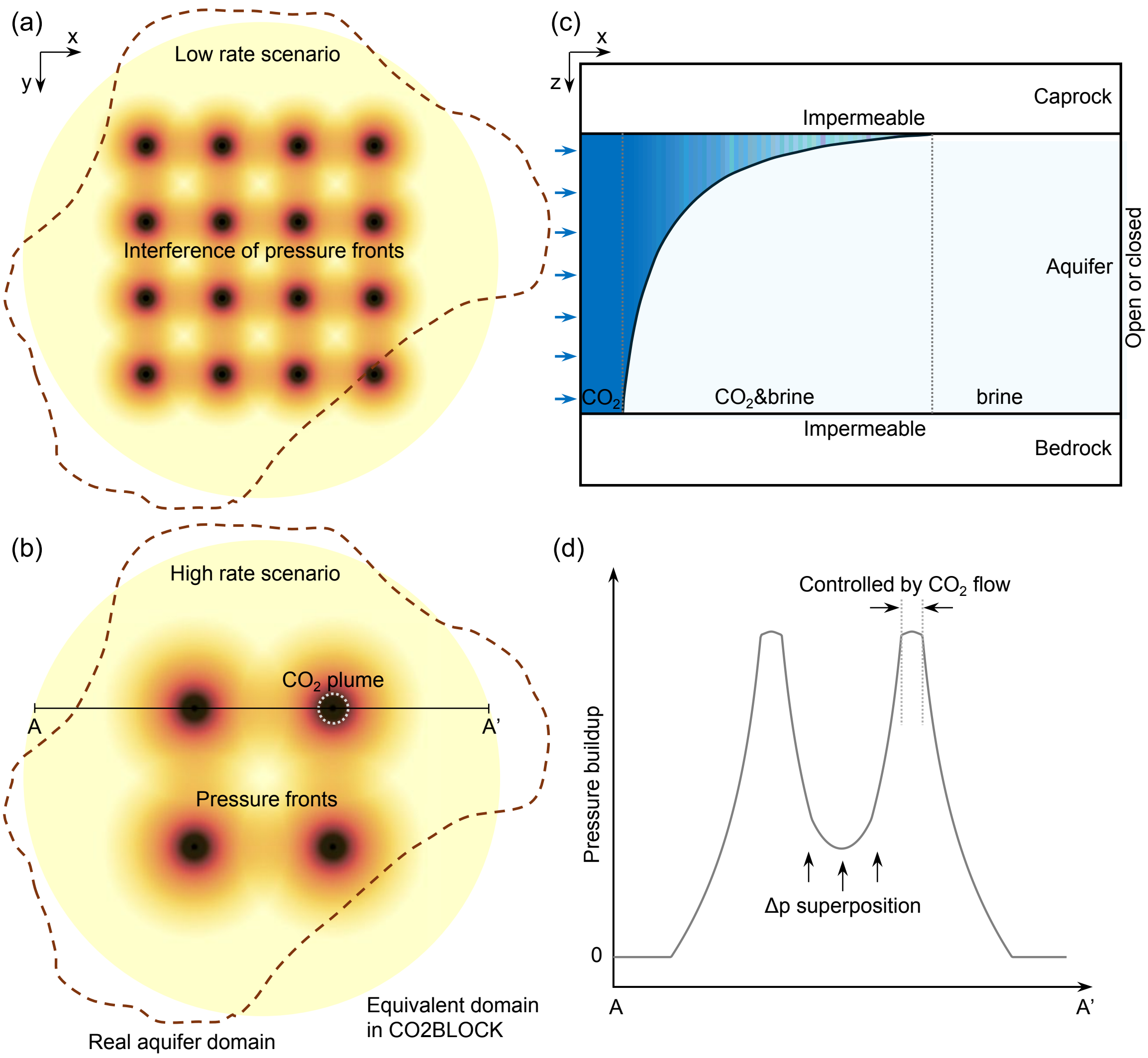

Fig. S1. Schematic illustration of the CO2BLOCK framework for estimating pressure-limited storage resources. (a,b) Examples of low- and high-rate injection scenarios illustrating the trade-off between the number of injection sites and their spacing. The dotted circle in panel (b) shows the extent of the CO_2 plume. CO2BLOCK evaluates sustainable storage resources across a range of site configurations. (c) Boundary flow conditions considered in the analytical pressure model for individual injection sites. (d) Analytical pressure profiles around injection sites, accounting for pressure interference using the superposition principle.

S1.1. Pressure buildup under open boundary flow conditions

The analytical model used in CO2BLOCK is adapted from Nordbotten et al. (2005) for CO_2 injection at a constant rate into an infinite, homogeneous, horizontal aquifer of constant thickness. The infinite-acting reservoir mimics open-flow boundary conditions. Injection is uniformly distributed across the entire height of the reservoir. Nordbotten et al. (2005) derived closed-form expressions for vertically averaged pressure build-up, assuming (1) instantaneous vertical equilibration of the phases under CO_2 gravity override, (2) a sharp CO_2-brine interface with each side of the interface fully saturated with the respective fluid, and (3) constant fluid densities and viscosities for each phase. These expressions specify three reservoir regions with distinct pressure trends: (1) adjacent to the wellbore, fully saturated by CO_2, (2) a transition region where the two fluids coexist, and (3) the far-field, fully saturated by brine (Nordbotten et al., 2005; Vilarrasa et al., 2010). This solution can be further simplified by replacing the transition zone with a fictitious equivalent vertical interface, which yields (De Simone et al., 2019)

$$\Delta p^{\infty}(r,t) = \frac{Q\mu_b}{2\pi k H \rho_c} \times \Delta p^{*}(t,r), \qquad \text{Eq. (S1)}$$

where

$$\Delta p^{*}(t,r) = \begin{cases} \frac{\mu_c}{\mu_b} \ln\left(\frac{\psi}{r}\right) + \ln\left(\frac{R}{\psi}\right) & , r \leq \psi \\ \ln\left(\frac{R}{r}\right) & , \psi < r \leq R \\ 0 & , R < r. \end{cases} \qquad \text{Eq. (S2)}$$

In these expressions, $\Delta p^{\infty}(r,t)$ is the pressure change at time t and distance r from the wellbore under open flow boundary conditions, Q is the mass flow rate, k is the absolute reservoir permeability, H is the reservoir thickness, ρ_c is the average CO_2 density across the plume, μ_b and μ_c are the average brine and CO_2 dynamic viscosities, respectively, $R =$

$\sqrt{2.25kt/(\mu_b(c_r + \phi c_b))}$ is the radius of influence over which pressure diffusion propagates at time t, ϕ is the rock porosity, c_r and c_b are rock and brine compressibilities, respectively, and ψ is the radius of the fictitious equivalent vertical interface and is given by

$$\psi = \xi e^{\theta}, \qquad \text{Eq. (S3)}$$

where $\xi = \sqrt{Qt/(\pi\phi H\rho_c)}$ is the extent of advective propagation of the cylindrical CO_2 front and $\theta = (\mu_c + \mu_b)/(\mu_c - \mu_b)\ln\left(\sqrt{\mu_c/\mu_b}\right) - 1$ represents a function of fluid mobilities. The density and viscosity of the fluids are estimated at the mean reservoir pressure, temperature, and salinity.

The performance of this simplified solution has been verified against numerical simulations (De Simone et al., 2019). Pore pressure varies logarithmically with distance from the injection wellbore. Thus, plotting pore pressure versus the logarithm of distance results in a bilinear curve with a sharp change in slope due to the fluid viscosity change at $r = \psi$. The original Nordbotten et al. (2005) solution returns the same estimates of pore pressure in the near-wellbore and far-field regions, but slightly different estimates in the transition zone as fluid viscosity is represented by a linear weighted average between those of brine and CO_2. We here use the compacted version of the solution (De Simone et al., 2019) as it simplifies calculations in a straightforward manner.

S1.2. Multi-site injection configurations

For multi-site injection, the pressure response at any location is obtained by applying the principle of linear superposition to the individual well solutions. To this end, Eqs. 1-3 are evaluated independently for each injection site using the corresponding distance to the observation point, and the resulting pressure contributions are summed to obtain the total

pressure buildup. For two-phase flow, which is inherently a non-linear phenomenon, the superposition approaximation results in an error whose magnitude grows with the number of injection sites (De Simone et al., 2019). The error arises because the pressure solution associated with each injection site is evaluated independently, neglecting the increased fluid mobility produced by CO_2 plumes around neighbouring injection sites. Consequently, pressure propagation in the far-field is no longer controlled solely by brine viscosity, as assumed in Eq. S2. Instead, the presence of intervening CO_2 plumes locally increase fluid mobility, reducing hydraulic resistance and attenuating pressure buildup. Neglecting this effect causes the superposition approach to systematically overestimate pressure buildup, resulting in conservative estimates of storage complex integrity.

S1.3. Extension to closed boundary flow conditions

For closed boundary flow conditions, pressure diffusion is confined within a finite aquifer volume and the injected CO_2 is accommodated by compression of the resident brine and the rock framework. In the initial version of CO2BLOCK, closed-boundary effects were approximated by adding a finite-domain correction factor to the dimensionless pressure response of the single-site Nordbotten solution (Eq. 2). The correction factor f_{cb} evolves with time as (De Simone and Krevor, 2021)

$$f_{\mathrm{cb}}(t) = \frac{2R^2}{2.25R_c^2} - \frac{3}{4}, \qquad \text{Eq. (S4)}$$

where R_c is the radius of the equivalent circular closed reservoir and time enters the expression through the radius of influence R. Inspired by pseudo-steady-state, single-phase radial flow in a closed reservoir (Zimmerman, 2018), this correction can provide a reasonable approximation for a single injection site located at the centre of a circular closed reservoir. However, its direct

application to multi-site injection configurations incurs error in pressure estimates. This is because each injection site is treated as if it were centred in its own equivalent closed reservoir, whereas in reality all sites pressurise a single bounded storage volume. In other words, direct superposition of single-site closed-boundary corrections virtually expands the pressurised reservoir volume, rather than treating the aquifer as one shared closed system. As a result, the superposed pressure field is not effectively constrained by the reservoir-wide material balance, resulting in an underestimation of pressure buildup. This underestimation translates into an overestimation of reservoir injectivity and, thus, larger estimates of pressure-limited CO_2 storage resources.

To resolve this issue, in CO2BLOCK-GROWTH, we reformulate the closed-boundary pressure response by adapting the decomposition concept proposed for decline curve analysis in multi-well hydrocarbon production fields (Marhaendrajana and Blasingame, 2001). This concept relies on the notion that a multi-well system can be represented by the aggregated response of a single well in a closed system only when the total material balance of the reservoir is enforced. Accordingly, we decompose the pressure field into a spatially averaged bulk-pressure component, $\Delta\bar{p}(t)$, and a zero-mean dynamic component, $\Delta p'(r,t)$, as

$$\Delta p(r,t) = \Delta\bar{p}(t) + \Delta p'(r,t). \qquad \text{Eq. (S5)}$$

By definition,

$$\Delta\bar{p}(t) = \frac{1}{A}\int_{\Omega} \Delta p(r,t)\, dA, \qquad \text{Eq. (S6)}$$

and therefore

$$\frac{1}{A}\int_{\Omega} \Delta p'(r,t)\, dA = 0. \qquad \text{Eq. (S7)}$$

The average pressure term represents the uniform reservoir-scale pressure increase consistent with the global material balance equation. For a total mass injection rate $Q_{\text{tot}} = NQ$, where N is the number of injection sites, this term is calculated as

$$\Delta\bar{p}(t) = \frac{Q_{\text{tot}} t}{\rho_c A H (c_r + \phi c_b)}, \qquad \text{Eq. (S8)}$$

where A is the reservoir area. This bulk-pressure term is applied only once for the entire reservoir as all injection sites share the same closed compressible volume.

On the other hand, the dynamic pressure component is approximated using the open-boundary Nordbotten solution (Eqs. S1-S3) after removal of its spatial mean over the actual closed reservoir domain Ω, which writes as

$$\langle \Delta p_i^{\infty} \rangle_{\Omega} = \frac{1}{A} \int_{\Omega_i^{eff}} \Delta p_i^{\infty}(r,t)\, dA, \qquad \text{Eq. (S9)}$$

where Ω_i^{eff} denotes the pressure-influence region of site i that lies inside the actual reservoir domain (Fig. S2). It is then straightforward to write the zero-mean local contribution from site i to the pressure change as

$$\Delta p_i'(r,t) = \Delta p_i^{\infty}(r,t) - \langle \Delta p_i^{\infty} \rangle_{\Omega}. \qquad \text{Eq. (S10)}$$

This pressure component accounts for the dynamics of pressure evolution around each individual injection site. The total pressure buildup under closed boundary flow conditions is finally calculated as

$$\Delta p(r,t) = \frac{Q_{\text{tot}} t}{\rho_c A H (c_r + \phi c_b)} + \sum_{i=1}^{N} \Delta p_i'(r,t). \qquad \text{Eq. (S11)}$$

The proposed formulation provides a computationally efficient approximation for closed-boundary flow conditions. It enforces the correct reservoir-scale material balance while retaining local pressure interference through superposition of the zero-mean dynamic pressure

contributions. Further improvements could be achieved by deriving exact closed-domain Green's functions for multiphase flow.

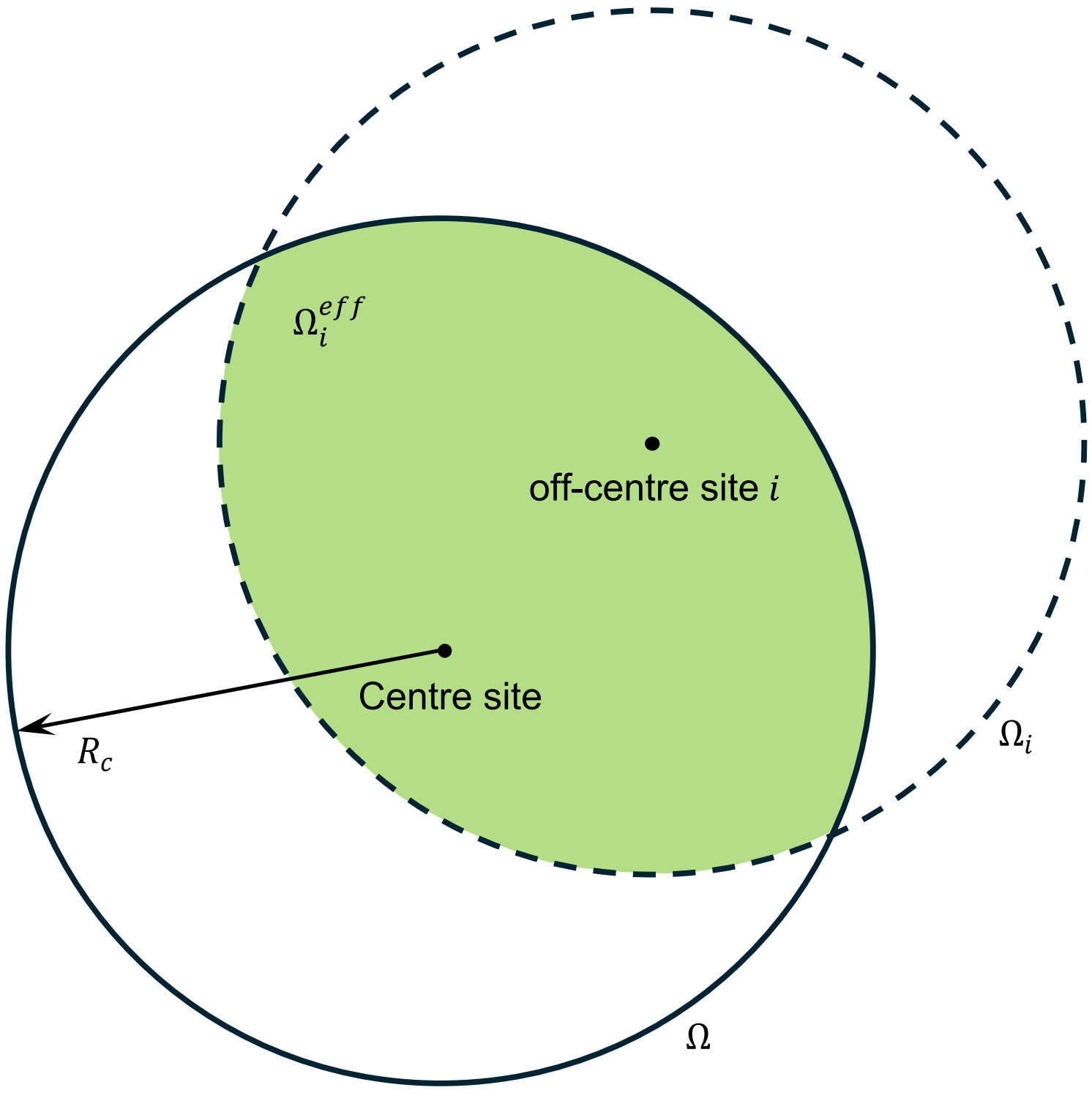

Fig. S2. Schematic illustration of the effective pressurised domain Ω_i^{eff} associated with an off-centre injection site under closed boundary conditions. The effective domain is the area over which the contribution of site i to pressure buildup is integrated to compute the spatially averaged pressure contribution that is subtracted from the dynamic pressure term. This area corresponds to the intersection between the pressure domain of injection site i (dashed circle) and the actual closed reservoir domain (solid circle).

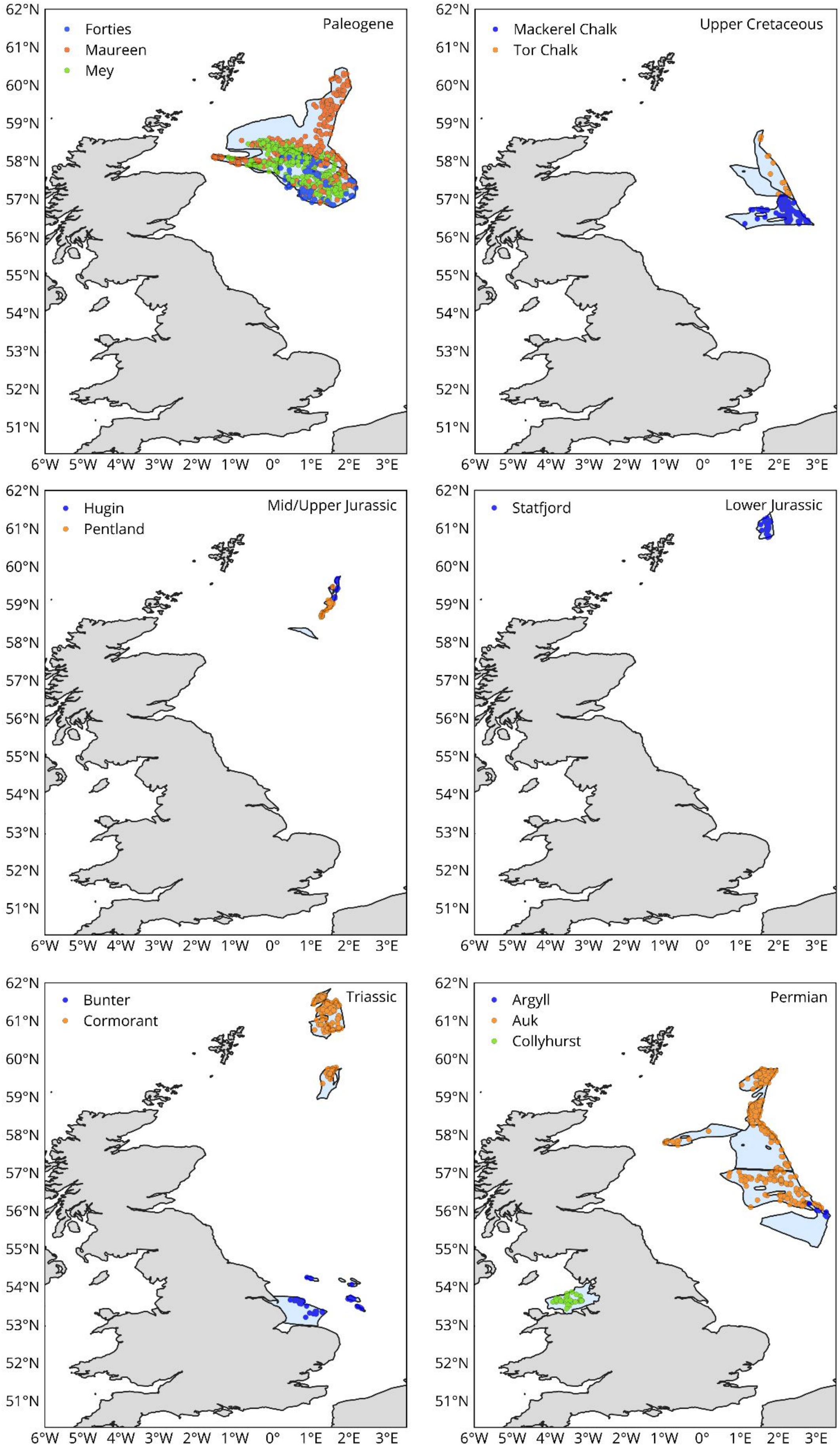
Paleogene
Forties
Maureen
Mey
Upper Cretaceous
Mackerel Chalk
Tor Chalk
Mid/Upper Jurassic
Hugin
Pentland
Lower Jurassic
Statfjord
Triassic
Bunter
Cormorant
Permian
Argyll
Auk
Collyhurst
62°N
61°N
60°N
59°N
58°N
57°N
56°N
55°N
54°N
53°N
52°N
51°N
6°W 5°W 4°W 3°W 2°W 1°W 0° 1°E 2°E 3°E

Fig. S3. Spatial distribution of wellbores in UK offshore CO_2 storage aquifers by geological age.

S2. Parameterisation of the geophysical model for the UK storage units

Saline aquifers are usually not extensively characterised, and uncertainties in parameters can result in order of magnitude variations in dynamic estiamtes of the storage resource base (De Simone and Krevor, 2021). We parameterise our calculations based primarily on data reported in the CO_2Stored database (Bentham et al., 2014; Energy Technologies Institute, 2018). The database provides likely values for geometrical parameters such as formation areal extent and thickness, as well as hydraulic characteristics such as rock porosity, permeability, compressibility and boundary flow conditions. Yet, these parameters may bear uncertainty to varying extents in estimates of storage formation pressure response to CO_2 injection. In particular, rock permeability is known to vary across spatial scales. While addressing scale-dependent permeability of the rock mass entails site-specific studies, we explore sensitivity of injection-induced pressure buildup to uncertainty in the permeability field by considering three alternative models where the permeability, k, is conservatively reduced by factors of 2, 5 and 10 –referred to as $k/2$, $k/5$ and $k/10$ models.

Hydraulic and geometrical parameters vary substantially across the selected UK storage units (Fig. S4). Average reservoir porosity spans a range of roughly 0.15, and permeability ranges over several hundred millidarcies. While these hydraulic attributes exhibit largely random spatial distributions, the geometrical characteristics of the storage units display meaningful regional trends across the four UK offshore sectors. In particular, the central and northern North Sea show markedly greater variability. These regions host several laterally extensive and thick storage units, with some exceeding 1 km in thickness in the Northern basin.

Furthermore, they are systematically deeper, with most units located below 2 km and some extending to ~3.5 km. These trends indicate that central and northern basins may offer considerable storage potential, although their development is likely to be more technically and economically demanding.

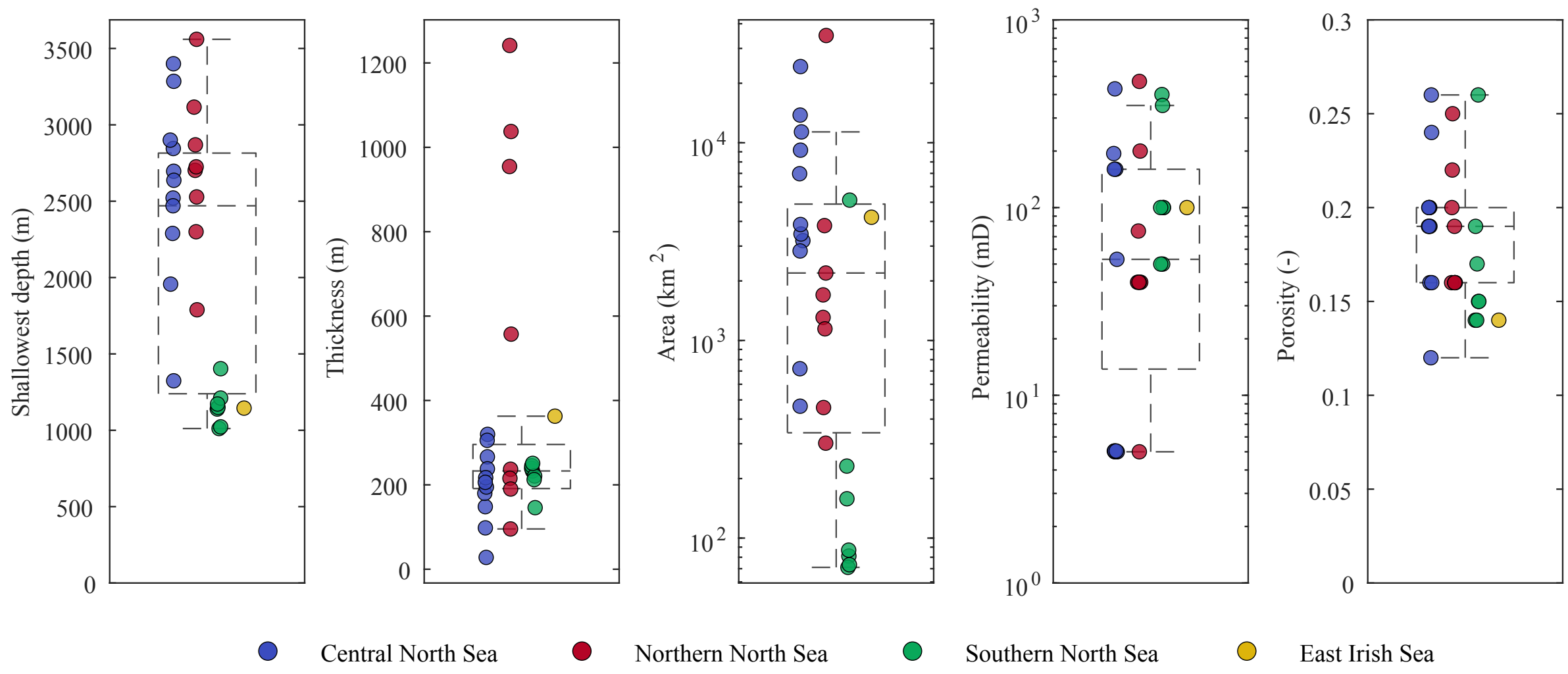

Fig. S4. Boxplot for a number of key parameters controlling reservoir pressure response to CO_2 injection, including shallowest reservoir depth, its thickness, areal extent, intrinsic permeability and porosity. From bottom to top, the box indicates the first quartile, median and third quartile of the data. Whiskers represent the minimum and maximum values, excluding outliers. Outliers are defined as data residing outside the range defined by 1.5 times the interquartile range beyond the first and third quartiles.

In contrast to hydraulic properties, information about geomechanical parameters is commonly lacking as they are more difficult to estimate. These parameters determine the susceptibility of the storage unit to mechanical failure in response to injection pressure. We conservatively assume that the formation is cohesionless and is crossed by optimally oriented faults to slip at the location of the largest pressure buildup across each study area. We adopt a representative fault friction coefficient of 0.6 (Jaeger et al., 2009). Eventually, the largest uncertainty is introduced by the state of stress, particularly, the ratio of the minimum to

maximum effective principal stress $S = \sigma'_3/\sigma'_1$, hereinafter referred to as the stress ratio, which imposes first-order control on reservoir integrity calculations. While initial reservoir pressure and overburden stress are reported for candidate storage units in the CO_2Stored database, we use public stress records at the regional scale to constrain the stress ratio for the regions of interest.

The North Sea is a relatively stable intraplate sedimentary basin, generally regarded as tectonically relaxed compared with other continental margins (Edwards et al., 1998). As a result, its present-day stress field is dominated by a tensile (normal) regime in the southern and central sectors, where the vertical stress, σ_v, slightly exceeds the maximum and minimum horizontal components, σ_H and σ_h, respectively, and horizontal stress anisotropy remains low, i.e., $\sigma_v > \sigma_H \approx \sigma_h$ (Fig. S5; Hillis and Nelson, 2005; Williams et al., 2015; Fellgett et al., 2018). Direct measurements of the minimum horizontal stress, σ_h, from more than 1000 (extended) leak-off tests indicate relatively high stress ratios, with a representative value of 0.78 in the southern North Sea and ranging between 0.5 and 0.9 under variable pore pressure, with an average of 0.71 in the central basin (Edwards et al., 1998). Toward the northern North Sea, the stress regime transitions to a more compressional state ($\sigma_H > \sigma_v \approx \sigma_h$), indicative of strike-slip to reverse faulting conditions (Fig. S5; Wiprut and Zoback, 2000; Hillis and Nelson, 2005). This transition is primarily attributed to deglaciation stress effects (Hillis and Nelson, 2005) and far-field compression associated with the Atlantic ridge push (Fejerskov and Lindholm, 2000). Estimates of the maximum horizontal stress, σ_H, remain sparse, as its determination is less straightforward than for other stress components. Extensive analyses of data from boreholes in the Visund field (Norwegian sector) suggest significant horizontal stress anisotropy with a factor of 1.25 (Wiprut and Zoback, 2000). Given that tectonic activity is less pronounced in the UK sector, this stress field likely represents a lower-bound estimate of the stress ratio in the northern North Sea and is adopted here for conservative estimates of storage

resources. A similar transition from strike-slip to reverse stress regimes seems to prevail in the East Irish Sea Basin (Williams et al., 2018). In the absence of sufficient data to contrain σ_H, we take the same stress field as in the northern North Sea for this region. Accordingly, we calculate stress ratios ranging from $S = 0.37$ to $S = 0.65$ with an average of $S = 0.53$ in these regions.

The vertical stress generally follows an average gradient of approximately 21 MPa/km. Reservoir pressures in the southern North Sea and East Irish Sea units are close to hydrostatic conditions, whereas pressure anomalies are observed in several units within the northern and central basins (Fig. S5). It is broadly demonstrated that horizontal stresses tend to increase with pressure abnormality (Zoback, 2010), reflecting the coupled response of the stress field to pore pressure variations. Thus, our estimates of the stress ratio, derived from minimum horizontal stress data in normally pressured settings, likely underestimate the true stress ratios and in this regard are conservative in the resource assessment. Units exhibiting elevated pressures are represented by Mohr circles located closer to the frictional failure envelope, indicating reduced capacity for CO_2 injection while preserving the mechanical stability of the storage complex (see inset in Fig. S5).

Across the whole studied region, the stress ration varies between 0.37 and 0.9 with an average of 0.66. The stress ratio can be written in terms of the mobilized friction coefficient, μ_{mob}:

$$\mu_{mob} = \frac{1-S}{2\sqrt{S}}, \qquad \text{Eq. (S12)}$$

which represents a measure of rock or optimally-oriented faults to failure. Failure takes place when the mobilized friction coefficient approaches the frictional strength of the rock. Data from the literature shows that the mobilized friction coefficient in sedimentary rocks broadly lie within the range from 0.2 to 0.6 (Vilarrasa and Carrera, 2015, Fig. S6). Mobilized friction

coefficients for the storage units in the studied region range from 0.05 to 0.52 with an average of 0.22, representing relatively large margin from failure conditions.

To assess the impact of uncertainties in the initial state of stress on geomechanical pressure limits, we systematically increase the initial maximum differential principal stress, $S_1 - S_3$, by factors of 1.1, 1.2, and 1.3. The comparatively well-constrained vertical stress is retained. Under normal-faulting conditions ($S_1 = S_v$), the increase in differential stress is introduced by reducing S_3. Under strike-slip conditions where both minimum and maximum principal stresses are horizontal, half of the prescribed increase is added to S_1 and half is subtracted from S_3. These scenarios progressively move the initial stress state towards the failure envelope (Fig. S6) and provide a conservative assessment of uncertainty in the geomechanical pressure limit.

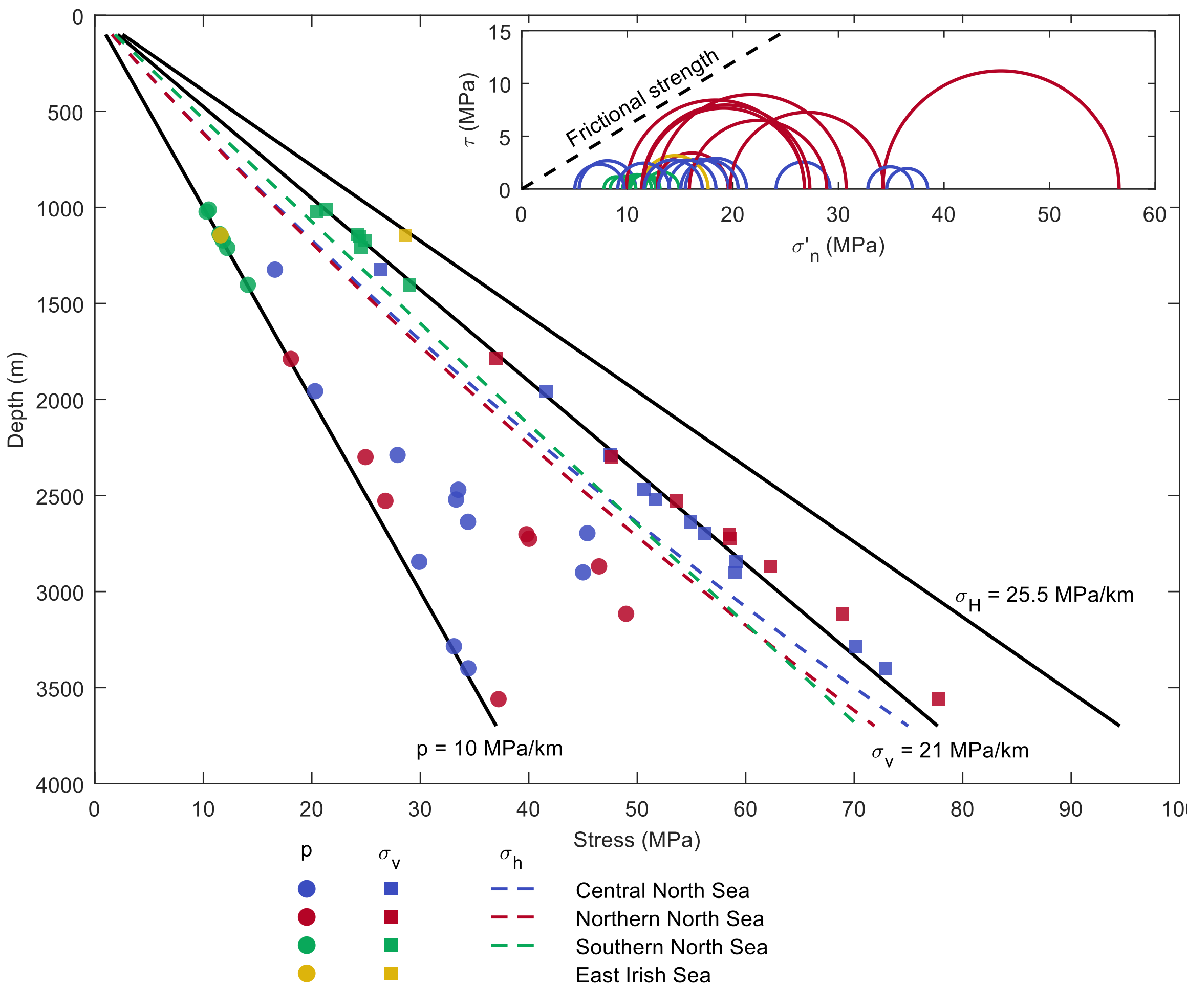

Fig. S5. In situ stress and pore pressure conditions in four UK offshore regions for CO_2 storage: central North Sea, northern North Sea, southern North Sea, and East Irish Sea. Average overburden stress (σ_v), hydrostatic pressure (p), measured minimum horizontal stress across the North Sea (σ_h) and maximum horizontal stress (σ_H) profiles are plotted as a function of depth (continuous lines), together with measured pore pressure and overburden stress at the shallowest depths of candidate reservoir units across the four regions (discrete symbols). The inset illustrates corresponding Mohr circles for 27 storage units, colored by region, and the frictional strength envelope ($\mu = 0.6$).

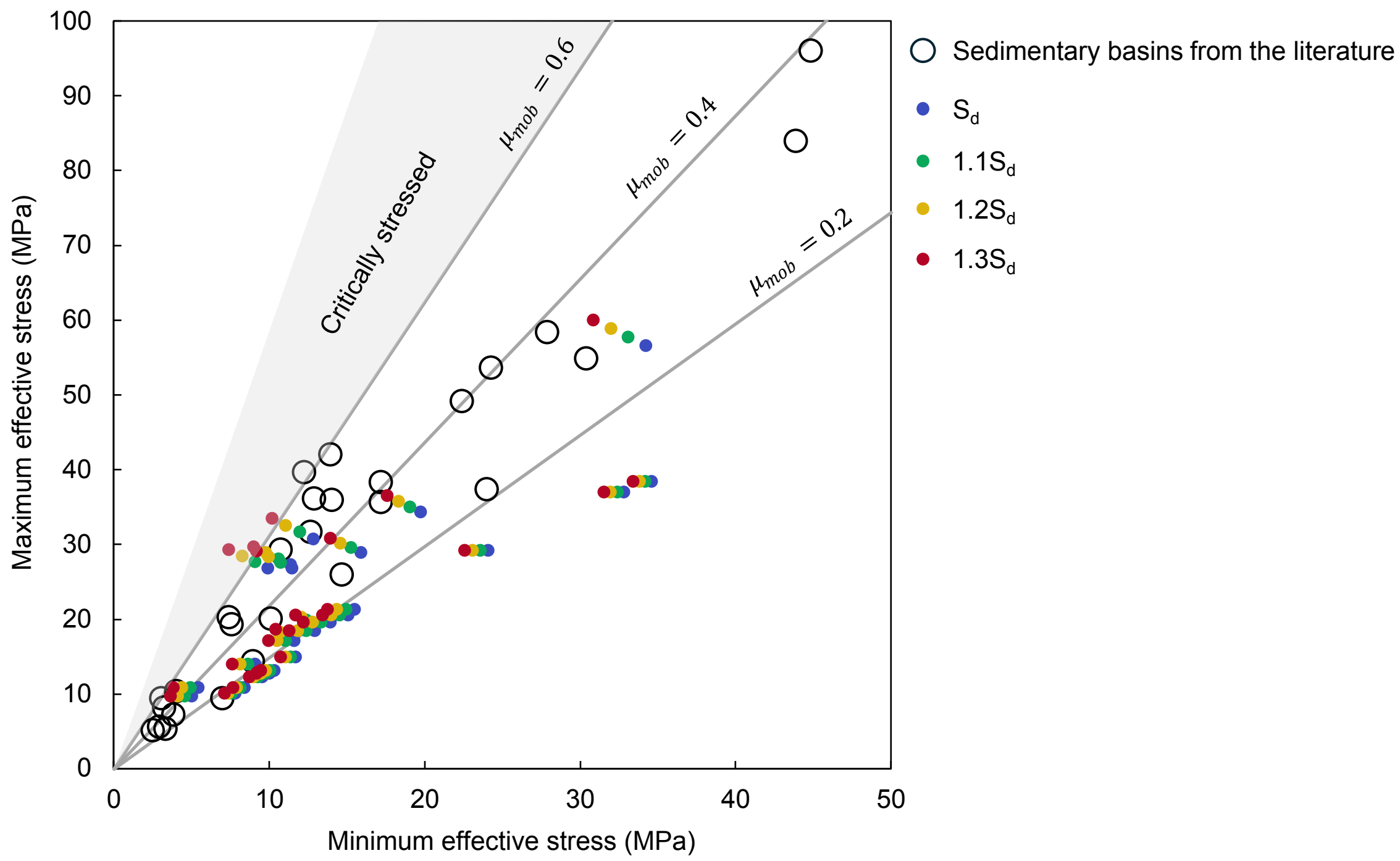

Fig. S6. Maximum versus minimum effective stress for data of sedimentary basins from the literature (Vilarrasa and Carrera, 2015) and the storage units in the North Sea considered in this study under the reference scenario constrained with field measurements (S_d) alongside three additional scenarios featuring enhanced differential stresses by factors of 1.1, 1.2 and 1.3 ($1.1S_d$-$1.3S_d$, respectively).

S3. Injectivity analysis of UK offshore storage units

Simulation results show that reservoir injectivity, defined as the achievable injection rate per unit increase in reservoir pressure, is governed jointly by reservoir properties, boundary flow conditions, and the spatial configuration of injection sites. In scenarios with few injection sites, where lateral pressure interference between individual sites is minimal, per-site injectivity is primarily controlled by intrinsic reservoir characteristics. In a storage unit like Auk 029 15 with relatively low intrinsic rock permeability (~5 mD) and closed flow boundaries, despite its large thickness (180 m) and large areal extent (~11,000 km^2), as well as favourable stress conditions (high stress ratio of 0.71), injection rate per site is limited to nearly 1.2 Mt yr^{-1} to maintain mechanical integrity (Fig. S7a). In contrast, Mey 5 exhibits high permeability (>400 mD), substantial thickness (~220 m), and open boundary conditions over a very large area (~24,000 km^2), which enables per-site injection rates as large as the model-imposed upper limit of 20 Mt yr^{-1} for up to ~12 injection sites without exceeding the critical pressure (Fig. S7b).

Feasible injection scenarios are principally constrained by the reservoir surface area, which imposes an upper bound on the allowable combinations of site number and spacing (blue lines in Fig. S7). The maximum per-site injectivity generally decreases with increasing number of injection sites across possible scenarios because of growing pressure interference resulting from shortening inter-site distances. Nevertheless, maximum storage capacity does not necessarily follow the same trend. For both example sites, the maximum cumulative storage capacity is achieved by deploying a large number of injection sites operating near the minimum per-site injection rate of 1 Mt yr^{-1} (Fig. S7). This trend shows that maximizing the total accessible reservoir area is generally more beneficial than maximizing injectivity at individual sites. Such dominance of total injection area over local injectivity loss is similarly observed in several other storage units with either open or closed boundaries (Table S1). However, smaller, closed-boundary reservoirs such as Hugin 009 18 and the Pentland units show markedly

different behaviour. In these cases, the sharp reduction in per-site injectivity associated with additional sites outweighs the benefit of increasing the number of injectors. As a result, the optimal strategy in these cases consists of deploying only a few high-rate injection sites (Table S1).

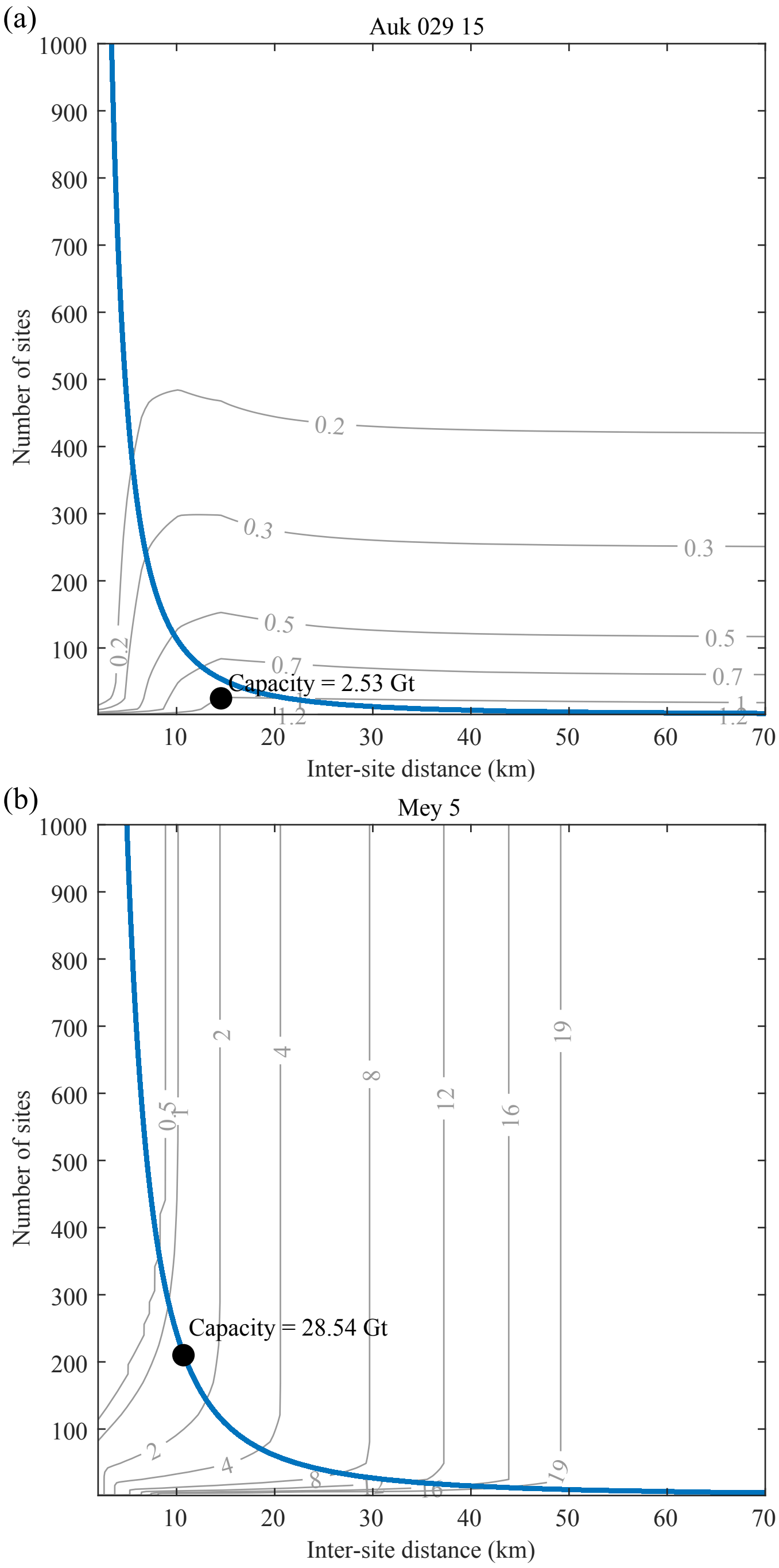

Fig. S7. Reservoir injectivity calculations for 100 years of continuous injection into (a) Auk 029 15 and (b) Mey 5 as representative examples of storage units with closed and open

boundary flow conditions, respectively, under different scenarios of injection patterns defined by the number and distance between the sites. Contours show injection rate per site (Mt yr^{-1}) and the blue line draws an upper bound limit to possible site numbers given the reservoir areal extent, i.e., possible scenarios reside to the left and below the blue line. The maximum storage capacity that can be achieved is shown by a filled circle.

Table S1. Maximum storage capacity, injection rate per site, and corresponding number and distance of sites required to achieve these rates for the selected UK storage locations. Calculations assume 100 years of continuous injection and a prescribed minimum intersite spacing of 2 km. Note that storage capacities denoted by "-" imply units that cannot maintain injection rates above the minimum threshold of 1 Mt yr^{-1} for 100 years even with one injection site.

Unit name	Capacity (Gt)	Rate per site (Mt yr^{-1})	Site number	Distance (km)	Boundary
Argyll 038 14	0.93	1.04	9	27.8	Closed
Auk 009 28	0.42	1.05	4	15	Closed
Auk 020 05	-	0.86	1	2	Closed
Auk 022 13	-	0.33	1	2	Closed
Auk 029 15	2.53	1.01	25	14.5	Closed
Bunter Closure 28	0.14	1.37	1	2	Closed
Bunter Closure 3	-	0.50	1	2	Closed
Bunter Closure 35	0.11	1.13	1	2	Closed
Bunter Closure 36	-	0.64	1	2	Closed
Bunter Closure 37	-	0.87	1	2	Closed
Bunter Closure 39	-	0.79	1	2	Closed
Bunter SS Fm Zone 1	4.99	1.19	42	11	Open
Collyhurst SS Fm 1	4.87	1.16	42	9.9	Closed
Cormorant 003 02	0.54	1.35	4	18	Closed
Cormorant 009 18	7.07	1.1	64	5.1	Closed
Cormorant 211 12	0.55	1.38	4	10.6	Closed
Cormorant 211 23	1.28	1.06	12	13.4	Closed
Forties 5	1.90	1.19	16	29.3	Open
Hugin 009 18	0.55	1.37	4	8.7	Closed
Mackerel Chalk 022 15	2.17	1.08	20	11.9	Open
Maureen 2	14.03	1.28	110	17.7	Open
Mey 5	28.54	1.36	210	10.7	Open
Pentland 009 28	2.46	2.74	9	8.9	Closed
Pentland 016 21b	1.02	2.54	4	10.7	Closed
Statfjord 211 23	0.22	1.1	2	23.8	Closed
Tor Chalk 022 018	4.56	1.09	42	9	Open
Tor Chalk 022 09	-	0.89	1	2	Open

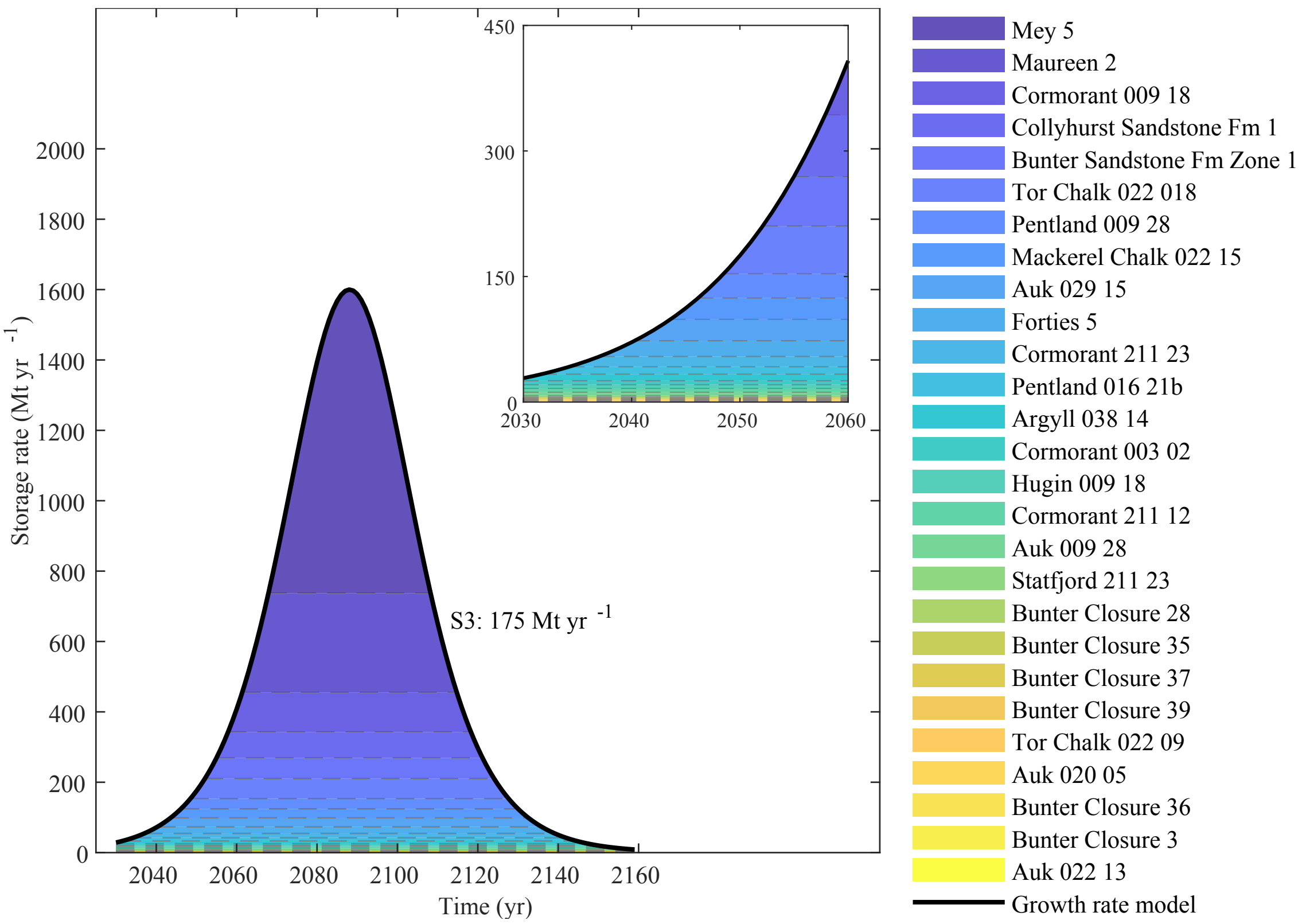

Fig. S8. Resource allocation under the growth rate model targeting a 2050 storage rate of 175 Mt yr^{-1} and a cumulative storage amount of 69 Gt, for the scenario prioritising units with smallest storage capacities. Inset shows zoomed view of the plot during the early stages of injection. Labels on the plot show the number of sites required to address the growth trajectory.

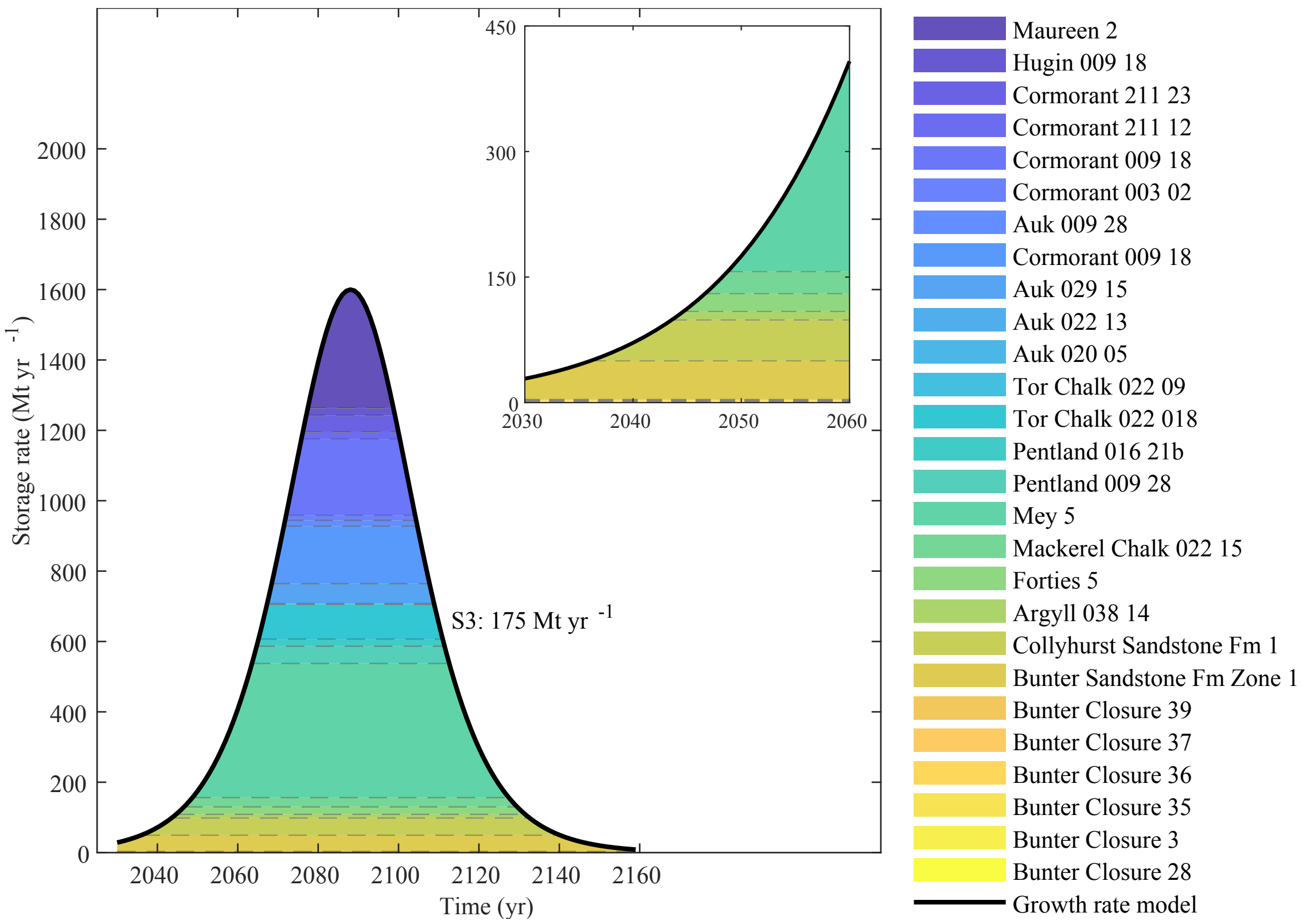

Fig. S9. Resource allocation under the growth rate model targeting a 2050 storage rate of 175 Mt yr^{-1} and a cumulative storage amount of 69 Gt, for the scenario consistent with planned projects, prioritising units in the southern North Sea, East Irish Sea, central North Sea, and northern North Sea, respectively. Inset shows zoomed view of the plot during the early stages of injection.

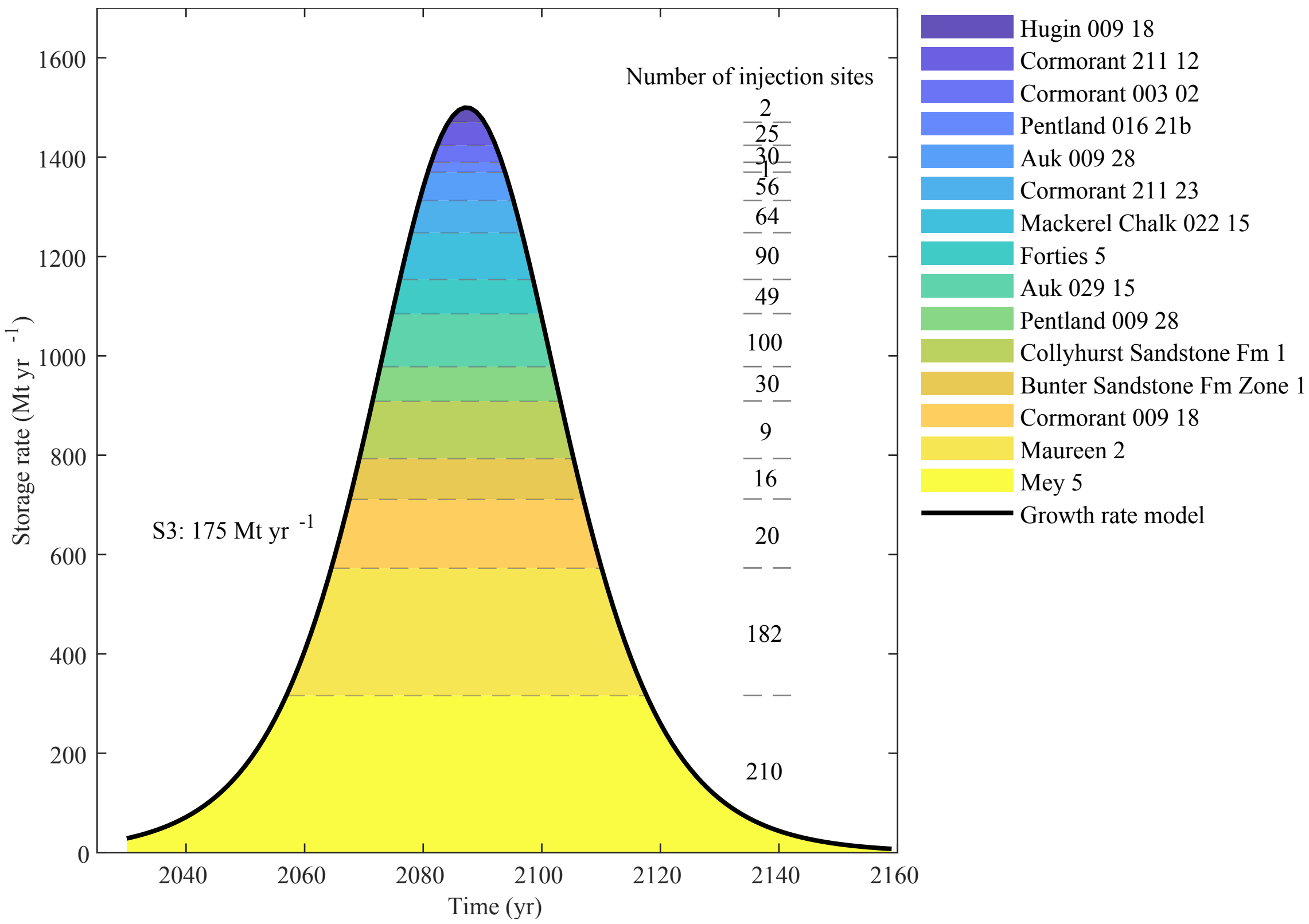

Fig. S10. Resource allocation for the growth rate model with a 2050 storage rate target of 175 Mt yr^{-1} and a cumulative storage amount of 64 Gt, when the maximum possible number of storage sites is constrained to historically drilled hydrocarbon wells in each region.

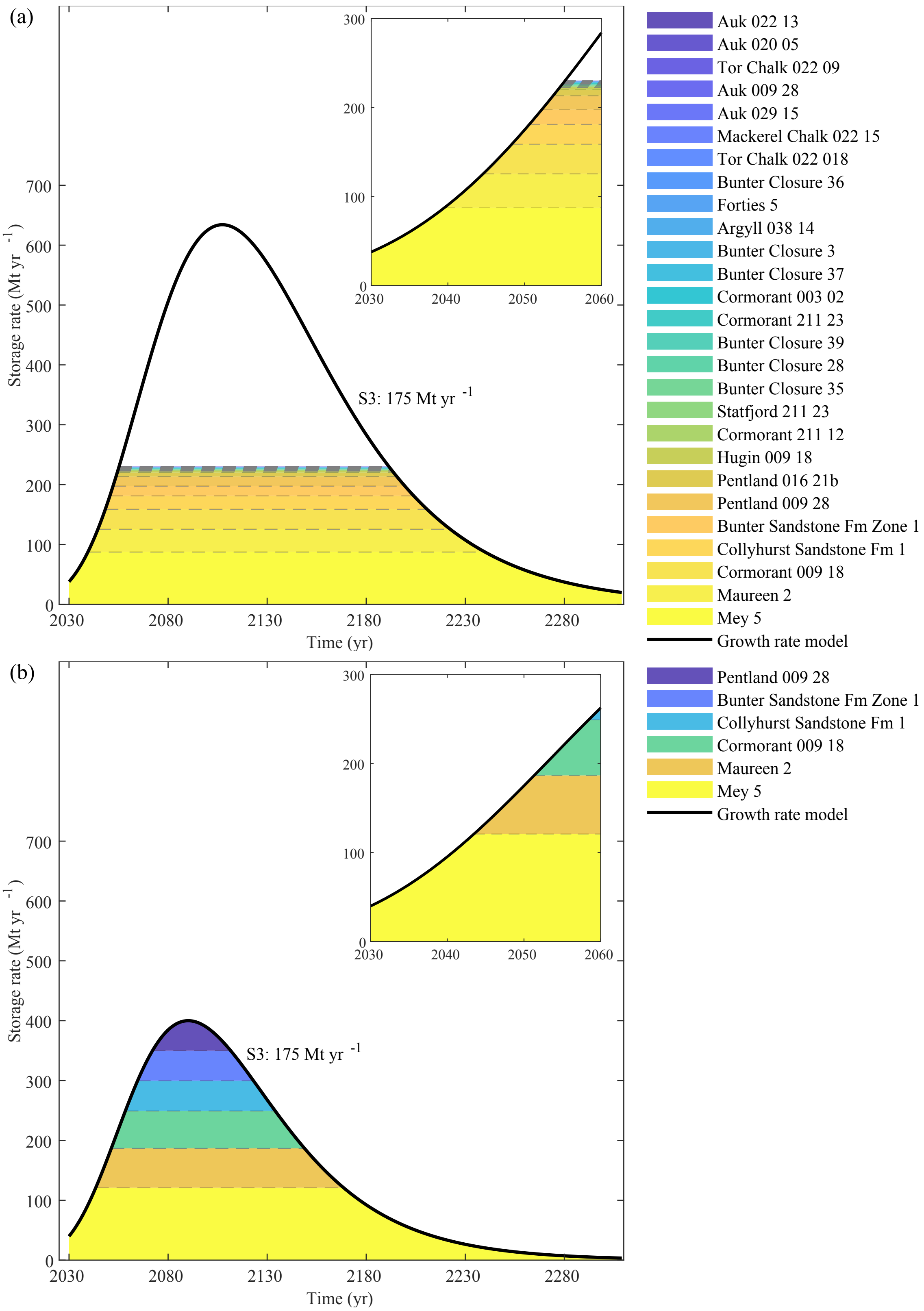

Fig. S11. Resource allocation for the Gompertz model targeting a CO_2 storage rate of 175 Mt yr^{-1} by 2050 and a (a) cumulative storage mass of 78 Gt, and (b) cumulative storage mass of 41 Gt, both considering reduced reservoir permeability by an order of magnitude ($k/10$

scenario). Comparison between the two panels shows how uncertainties in the permeability could affect addressing long-term scaleup trajectories. Insets show zoomed views of the plots during the early stages of injection.

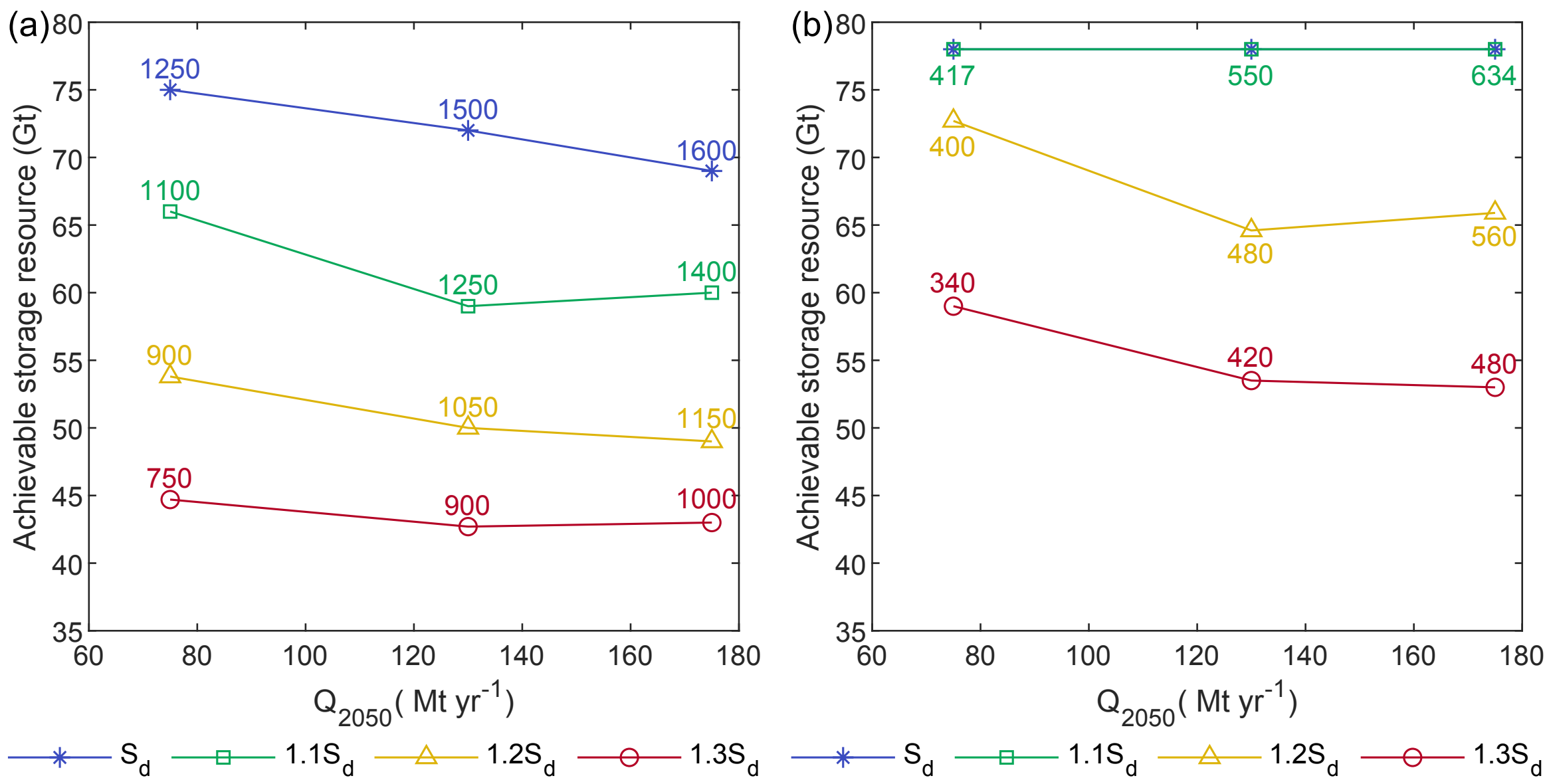

Fig. S12. Estimated achievable storage resource for (a) logistic and (b) Gompertz growth trajectories targeting 75, 130, and 175 Mt yr^{-1} by 2050, considering uncertainties in the initial state of stress: the reference stress state estimated from regional stress measurements (S_d) and conservative scenarios with differential stress increased by factors of 1.1, 1.2, and 1.3 ($1.1S_d$, $1.2S_d$, and $1.3S_d$, respectively). The corresponding peak injection rates (Mt yr^{-1}) for each scenario are shown as labels.

References

Bentham, M., Mallows, T., Lowndes, J., Green, A., 2014. CO_2 STORage evaluation database (CO_2 Stored). The UK's online storage atlas. Energy Procedia, 63, 5103-5113. https://doi.org/10.1016/j.egypro.2014.11.540

De Simone, S., Jackson, S.J., Krevor, S., 2019. The error in using superposition to estimate pressure during multi-site subsurface CO_2 storage. Geophys. Res. Lett. 46 (12), 6525–6533. https://doi.org/10.1029/2019GL082738

De Simone, S., Krevor, S., 2021. A tool for first order estimates and optimisation of dynamic storage resource capacity in saline aquifers. Int. J. Greenhouse Gas Control 106, 103258. https://doi.org/10.1016/j.ijggc.2021.103258

Edwards, S.T., Meredith, P.G., Murrell, S.A.F., 1998. An investigation of leak-off test data for estimating in-situ stress magnitudes: application to a basinwide study in the North Sea. In SPE/ISRM Rock Mechanics in Petroleum Engineering 1998 July 8 (pp. SPE-47272). SPE.

Energy Technologies Institute, 2018. Options, Choices, Actions. https://www.eti.co.uk/options-choices-actions-2018/

Fejerskov, M., Lindholm, C., 2000. Crustal stress in and around Norway: an evaluation of stress-generating mechanisms. In Dynamics of the Norwegian Margin. Nøttvedt, A. https://doi.org/10.1144/GSL.SP.2000.167.01.19

Fellgett, M.W., Kingdon, A., Williams, J.D. and Gent, C.M., 2018. Stress magnitudes across UK regions: New analysis and legacy data across potentially prospective unconventional resource areas. Mar. Pet. Geol. 97, 24-31. https://doi.org/10.1016/j.marpetgeo.2018.06.016

Hillis, R.R. and Nelson, E.J., 2005. In situ stresses in the North Sea and their applications: Petroleum geomechanics from exploration to development. In Petroleum Geology: North-West

Europe and Global Perspectives—Proceedings of the 6th Petroleum Geology Conference, A. G. Doré, B. A. Vining. https://doi.org/10.1144/0060551

Jaeger, J.C., Cook, N.G., Zimmerman, R., 2009. Fundamentals of rock mechanics. John Wiley & Sons.

Kivi, I.R., De Simone, S., Krevor, S., 2025. A simplified physics model for estimating subsurface CO_2 storage resources constrained by fault slip potential. J. Rock Mech. Geotech. Eng. 18(4), 2546-2560. https://doi.org/10.1016/j.jrmge.2025.06.031

Marhaendrajana, T., Blasingame, T.A., 2001. Decline curve analysis using type curves—evaluation of well performance behavior in a multiwell reservoir system. In SPE Annual Technical Conference and Exhibition? (pp. SPE-71517). SPE.

Nordbotten, J.M., Celia, M.A., Bachu, S., 2005. Injection and storage of CO_2 in deep saline aquifers: analytical solution for CO_2 plume evolution during injection. Transp. Porous Med. 58 (3), 339–360. https://doi.org/10.1007/s11242-004-0670-9

Vilarrasa, V., Bolster, D., Dentz, M., Olivella, S., Carrera, J., 2010. Effects of CO_2 compressibility on CO_2 storage in deep saline aquifers. Transp. Porous Med. 85 (2), 619–639. https://doi.org/10.1007/s11242-010-9582-z

Vilarrasa, V., Carrera, J. 2015. Geologic carbon storage is unlikely to trigger large earthquakes and reactivate faults through which CO_2 could leak. Proc. Natl. Acad. Sci. U.S.A., 112(19), 5938-5943. https://doi.org/10.1073/pnas.1413284112

Williams, J.D.O., Fellgett, M.W., Kingdon, A., Williamson, J.P., 2015. In-situ stress orientations in the UK Southern North Sea: Regional trends, deviations and detachment of the post-Zechstein stress field. Mar. Pet. Geol. 67, 769-784. https://doi.org/10.1016/j.marpetgeo.2015.06.00

Williams, J.D.O., Gent, C.M.A., Fellgett, M.W. and Gamboa, D., 2018. Impact of in situ stress and fault reactivation on seal integrity in the East Irish Sea Basin, UK. Mar. Pet. Geol. 92, 685-696. https://doi.org/10.1016/j.marpetgeo.2017.11.030

Wiprut, D., Zoback, M.D., 2000. Fault reactivation and fluid flow along a previously dormant normal fault in the northern North Sea. Geology 28(7), 595-598. https://doi.org/10.1130/0091-7613(2000)28<595:FRAFFA>2.0.CO;2

Zimmerman, R. (2018). The Imperial College lectures in petroleum engineering. volume 5: Fluid flow in porous media. World Scientific Publishing Europe. https://doi.org/10.1142/q0146

Zoback, M.D., 2010. Reservoir geomechanics. Cambridge university press.
